# Do Early Infant Feeding Practices and Modifiable Household Behaviors Contribute to Age-Specific Interindividual Variations in Infant Linear Growth? Evidence from a Birth Cohort in Dhaka, Bangladesh

**DOI:** 10.1093/cdn/nzab077

**Published:** 2021-04-30

**Authors:** Sarah L Silverberg, Huma Qamar, Farhana K Keya, Shaila S Shanta, M Munirul Islam, Tahmeed Ahmed, Joy Shi, Davidson H Hamer, Stanley Zlotkin, Abdullah Al Mahmud, Daniel E Roth

**Affiliations:** Pediatrics Residency Program, BC Children's Hospital and University of British Columbia, Vancouver, British Columbia, Canada; Centre for Global Child Health, Hospital for Sick Children, Toronto, Ontario, Canada; Centre for Global Child Health, Hospital for Sick Children, Toronto, Ontario, Canada; Nutrition and Clinical Services Division, icddr,b, Dhaka, Bangladesh; Nutrition and Clinical Services Division, icddr,b, Dhaka, Bangladesh; Nutrition and Clinical Services Division, icddr,b, Dhaka, Bangladesh; Nutrition and Clinical Services Division, icddr,b, Dhaka, Bangladesh; Centre for Global Child Health, Hospital for Sick Children, Toronto, Ontario, Canada; Department of Global Health, Boston University School of Public Health and Section of Infectious Diseases, Department of Medicine, Boston University School of Medicine, Boston, MA, USA; Centre for Global Child Health, Hospital for Sick Children, Toronto, Ontario, Canada; Department of Pediatrics, Hospital for Sick Children and University of Toronto, Toronto, Ontario, Canada; Nutrition and Clinical Services Division, icddr,b, Dhaka, Bangladesh; Centre for Global Child Health, Hospital for Sick Children, Toronto, Ontario, Canada; Department of Pediatrics, Hospital for Sick Children and University of Toronto, Toronto, Ontario, Canada

**Keywords:** child growth, length-for-age *z*-scores, stunting, breastfeeding, infant feeding, risk factors, growth models, developing countries, Bangladesh

## Abstract

**Background:**

Causes of infant linear growth faltering in low-income settings remain poorly understood. Identifying age-specific risk factors in observational studies might be influenced by statistical model selection.

**Objectives:**

To estimate associations of selected household factors and infant feeding behaviors within discrete age intervals with interval-specific changes in length-for-age *z*-scores (LAZs) or attained LAZ, using 5 statistical approaches.

**Methods:**

Data from a birth cohort in Dhaka, Bangladesh (*n* = 1157) were analyzed. Multivariable-adjusted associations of infant feeding patterns or household factors with conditional LAZ (cLAZ) were estimated for 5 intervals in infancy. Two alternative approaches were used to estimate differences in interval changes in LAZ, and differences in end-interval attained LAZ and RRs of stunting (LAZ < −2) were estimated.

**Results:**

LAZ was symmetrically distributed with mean ± SD = −0.95 ± 1.02 at birth and −1.00 ± 1.04 at 12 mo. Compared with exclusively breastfed infants, partial breastfeeding (difference in cLAZ: −0.11; 95% CI: −0.20, −0.02) or no breastfeeding (−0.30; 95% CI: −0.54, −0.07) were associated with slower growth from 0 to 3 mo. However, associations were not sustained beyond 6 mo. Modifiable household factors (smoking, water treatment, soap at handwashing station) were not associated with infant growth, attained size, or stunting. Alternative statistical approaches yielded mostly similar results as conditional growth models.

**Conclusions:**

The entire infant LAZ distribution was shifted down, indicating that length deficits were mostly caused by ubiquitous or community-level factors. Early-infant feeding practices explained minimal variation in early growth, and associations were not sustained to 12 mo of age. Statistical model choice did not substantially alter the conclusions. Modifications of household hygiene, smoking, or early infant feeding practices would be unlikely to improve infant linear growth in Bangladesh or other settings where growth faltering is widespread.

## Introduction

Optimal fetal and infant growth is a foundation of child health and neurodevelopment (1). It has therefore been of longstanding global health concern that infants in low- and middle-income countries (LMICs) are generally shorter and have slower average growth velocities compared with healthy norms (2, 3). A common pattern of slow linear growth in LMICs is reflected at the population level by a low mean length-for-age *z*-score (LAZ) at birth and a further decline in mean LAZ during the first 2 y of life (2, 4). The population burden of linear growth faltering (i.e., decline in LAZ) is conventionally indicated by the stunting prevalence (i.e., proportion of children with LAZ < − 2). Although there has been a progressive decrease in the global prevalence of stunting in children aged <5 y, the prevalence in South Asia remains high (33% according to 2019 estimates) (5).

Numerous maternal, perinatal, infant, and household characteristics have been associated with childhood stunting, primarily in cross-sectional studies (1, 6). A systematic review identified birth outcomes [preterm and small-for-gestational-age (SGA)] and environmental factors (unimproved water and sanitation, biomass fuel use) as dominant clusters of individual-level risks to which stunting was attributed (6). Using a different evidence synthesis approach, others have concluded that diarrhea, poor dietary diversity, SGA, and biomass fuel use were the major contributors to stunting prevalence (7). However, relatively few longitudinal studies in LMICs have examined age-specific effects of modifiable household and early postnatal feeding practices on linear growth in infancy (8–13). Furthermore, a systematic review of early-life intervention trials found that although providing multiple micronutrient supplements to infants had a modest significant effect on LAZ, there were no convincing growth-promoting effects of other postnatal interventions (food supplements, deworming, maternal education, water/sanitation) (14). Negative results of many intervention studies have highlighted the concern that stunting risk factors identified in observational studies can be difficult to modify or that associations are confounded. Furthermore, aggregation or comparison of findings across longitudinal studies of infant growth risk factors is complicated by the use of a wide range of child growth models (15), the choice of which can influence the magnitude of observed exposure-growth associations (16).

To estimate the contribution of infant feeding patterns and selected modifiable household behaviors to age interval–specific variations in early infant growth in a low-resource setting, and to directly assess the effect of statistical model selection on inferences, we conducted a longitudinal observational study of linear growth from birth to 12 mo of age in a cohort of Bangladeshi infants. First, we described the overall mean LAZ trajectory and parameters of the LAZ distribution throughout infancy, in relation to international norms. Second, we analyzed the associations of selected modifiable household and early postnatal feeding factors with infant growth in discrete intervals using conditional growth models (“residuals method”) as the primary analytic. Four alternative growth modeling approaches were used to test the sensitivity of estimates and inferences to model choice.

## Methods

### Study design

This longitudinal cohort study was based on secondary analyses of data from the Maternal Vitamin D and Infant Growth (MDIG) trial, a randomized controlled trial assessing the effect of maternal vitamin D supplementation during pregnancy and lactation on infant growth, conducted from 2014 to 2018 in Dhaka, Bangladesh (trial registration number: NCT01924013) (17, 18). Inclusion criteria for the MDIG trial are described in **Supplemental Methods 1**. Household characteristics were collected at prenatal enrollment and at 9 mo postpartum. Parental anthropometry was conducted at enrollment and at 12 mo postpartum. Infant dietary history based on maternal/caregiver report was assessed weekly from birth until 6 mo of age. Infant anthropometric measures were scheduled at trimonthly intervals during infancy, with the addition of another measurement at 1 visit in the first 2 mo (randomly assigned to 2, 4, 6, or 8 wk after birth). Among live births in the MDIG trial (*n* = 1254), infants were eligible for inclusion in the present study if anthropometric data were available at ≥2 time points from birth to 12 mo of age. Approval for the MDIG trial was obtained from research ethics committees at the Hospital for Sick Children and the International Center for Diarrheal Disease Research, Bangladesh (icddr,b). Written informed consent was obtained from all mothers of infants for participation. The Strengthening the Reporting of Observational Studies in Epidemiology (STROBE) cohort reporting guidelines were used for this report (19).

### Outcome data collection and variable definitions

Crown–heel length was measured independently by 2 study personnel using standard length boards. Measurements were repeated if the difference between paired measurements was >7 mm. The length board that was initially used had a counter display and ball-bearing–mounted sliding footboard (Harpenden infantometer; Holtain); however, this was switched to a wooden length board (Infant/Child ShorrBoard; Weigh and Measure) because of frequent decalibration of the Harpenden length board. Of 1098 infants in this study with length data at birth, 276 (25%) were measured using the Harpenden length board. Similarly, of 1094 infants with length data at 3 mo, 104 (9.5%) were measured using the Harpenden length board.

Means of the final pair of measurements were used to generate age- and sex-standardized LAZ using a combination of growth standards: *1*) Intergrowth-21st Newborn Size Standards for measurements taken within 48 h of birth (20); *2*) Intergrowth-21st International Postnatal Growth Standards for Preterm Infants for infants born earlier than 37 wk of gestation and measured up to 64 wk postmenstrual age (21); and *3*) the WHO Child Growth Standards for term infants measured beyond birth and for preterm infants measured beyond 64 wk postmenstrual age (22). Length and LAZ were assessed for biological plausibility and temporal consistency and reconciled using the jackknife residuals method (23).

LAZs at 4 time points were included: birth, 3 mo, 6 mo, and 12 mo of age based on predefined criteria on age in days at the time of each measurement (**Supplemental Methods 2**). Growth in 5 age intervals was assessed: *1*) birth to 3 mo of age; *2*) 3 to 6 mo of age; *3*) birth to 6 mo of age; *4*) 6 to 12 mo of age; and *5*) birth to 12 mo of age. These intervals were chosen to identify the effects of feeding practices during the period in which exclusive breastfeeding is recommended (birth to 6 mo), and to assess if effects were sustained up to 12 mo of age. Because breastfeeding patterns change substantially during the first 6 mo, we further divided the birth to 6-mo exposure period into 2 intervals to enable estimation of separate effects of early compared with late feeding practices.

### Exposure data collection and variable definitions

#### Infant feeding patterns (birth to 3 mo or birth to 6 mo)

Study personnel collected maternal/caregiver recall of infant feeding history on a weekly basis from birth to 26 wk of age. Each child-week was classified according to WHO definitions as: exclusively breastfed (breast milk only); predominantly breastfed (breast milk with water, sugar water, honey, or other nonmilk, nonformula liquid); partially breastfed (breast milk with animal, powdered or condensed milk, and solid or semisolid foods); or not breastfed (24). For each growth interval, classification of each infant with respect to this breastfeeding status hierarchy was based on the least optimal breastfeeding category achieved in any observed week during that interval (Supplemental Methods 2). Infants with missing feeding data for both of the last 2 wk of an interval were “not able to be classified.” The duration of exclusive breastfeeding (EBF) was calculated as the number of continuous weeks from birth to 26 wk in which an infant was classified as exclusively breastfed. In the primary analyses, we counted the first week of life as EBF when deriving the classification of feeding pattern and calculating EBF duration, because prelacteal feeding was a common practice in this setting in otherwise exclusively breastfed infants. However, in sensitivity analyses, we included the first week in the determination of breastfeeding status and EBF duration. In additional sensitivity analyses, we used varying criteria to derive the breastfeeding pattern and duration of exclusive breastfeeding to assess the robustness of our findings (Supplemental Methods 2).

Infants were classified as ever or never having had exposure to an animal-source food or formula in the first 3 mo and first 6 mo of life. In primary analyses for each of these exposures, we included infants in the model if we had data on the exposure of interest (i.e., animal-source food or formula exposure in the first 3 or 6 mo of life) for at least half of the weeks in the observation period. In sensitivity analyses, we included all infants regardless of the number of weeks of data available (Supplemental Methods 2). Feeding-related data were collected in the first 26 wk; therefore, we considered lagged associations of feeding variables to 6 mo with growth outcomes from 6 to 12 mo. Mothers were also asked weekly from birth to 26 wk of age if they were concerned about their baby's feeding or weight gain. To address a potential mechanism of reverse causality (i.e., caregiver concern about poor growth leading to abandonment of EBF), we generated a derived variable representing any such “maternal concern” expressed in the month prior to a change from EBF to a different feeding pattern (e.g., to partial breastfeeding or no breastfeeding).

#### Household sanitation and smoking

Household sanitation was assessed using 2 separate variables ascertained in a household questionnaire at maternal prenatal enrollment. Drinking water treatment (yes/no) was self-reported by the mother, whereas presence of soap at the handwashing area (yes/no) was visually assessed by study personnel. Household tobacco smoking was ascertained at maternal prenatal enrollment. Type of toilet facilities (95% flush or pour water into a piped sewer/tank/latrine), water source (80% piped into house or lot), and cooking source/fuel (95% not cooking inside the house with solid fuel) were not pursued beyond preliminary analyses due to inadequate heterogeneity in the distribution of these variables.

#### Additional covariates

Potential confounders included maternal age, maternal height, 12-mo postpartum maternal BMI as a proxy of preconception BMI, maternal and paternal education levels, maternal and paternal occupations, wealth index, number and age of siblings, delivery mode and location, neonatal hospitalization, infant sex, infant weight-for-age *z*-score (WAZ), and weight-for-length *z*-score (WFL) at birth. Principal components analysis using household asset data was used to derive the wealth index, which served as a proxy measurement of socioeconomic status (18). The specific set of confounders included in each model depended on an a priori constructed directed acyclic graph (DAG) of hypothesized pathways (**Supplemental Figure 1**). Vitamin D supplementation group assignment and maternal height were included in all analyses.

### Statistical analysis

The LAZ distribution at the beginning of each age interval was described in terms of its mean, median, SD, kurtosis, and skewness. Normality was tested using the Kolmogorov–Smirnov test. To estimate associations between risk factors and linear growth, primary analyses used the conditional growth (residuals) method involving 2-stage linear regressions in which LAZ at the end of a given time interval was first regressed on the LAZ at the beginning of the interval:

(1a) $$\begin{eqnarray*}
{\rm{LA}}{{\rm{Z}}_{it}} = {\beta _0} + {\beta _1}{\rm{LA}}{{\rm{Z}}_{i,t - 1}} + {\varepsilon _{it}}
\end{eqnarray*}$$

Then, the infant-specific residuals from the first model (referred to as conditional LAZ, or cLAZ) were regressed on the specified risk factor of interest, X, and a set of covariates, C_1_, …, C_j_ based on the relevant DAG, in a second linear regression model:

(1b) $$\begin{eqnarray*}
{\varepsilon _{it}} = c{\rm{LA}}{{\rm{Z}}_{it}} = {\beta _0} + {\beta _1}{X_i} + {\beta _2}{C_{i1}} + \ldots + {\beta _{j + 1}}{{\rm{C}}_{{\rm{ij}}}} + \varepsilon _i^*
\end{eqnarray*}$$

In models addressing infant feeding exposures, we estimated associations of feeding status in the 0–3-mo period with concurrent growth from 0 to 3 mo, and lagged associations of feeding status in the 0–3-mo interval with growth in later intervals (3–6, 0–6, 0–12, and 6–12 mo). We also estimated associations of feeding from 0 to 6 mo on concurrent growth in the 0–3-, 0–6-, and 3–6-mo intervals, and lagged associations with growth in the 0–12- and 6–12-mo intervals.

Alternate modeling approaches were used in sensitivity analyses:

1. Baseline-adjusted attained size model (ANCOVA): LAZ at the end of a time interval, LAZ_t_, was regressed on the exposure of interest, a set of confounders, and LAZ at the beginning of the given time interval, LAZ_t-1_, using linear regression:
  (2) $$\begin{eqnarray*}
LA{Z_{it}} &=& {\beta _0} + {\beta _1}LA{Z_{i,t - 1}} + {\rm{ }}{\beta _2}X + {\beta _3}{C_{i1}}\nonumber\\
&& +\, \ldots + {\beta _{j + 2}}{C_{ij}} + {\varepsilon _i}
\end{eqnarray*}$$
2. Change score model: The change (Δ) in LAZ from the beginning to the end of a given interval, ΔLAZ_t_, was regressed on the specified risk factors of interest and covariates using linear regression:
  (3) $$\begin{eqnarray*}
{\rm{LA}}{{\rm{Z}}_{it}} - {\rm{LA}}{{\rm{Z}}_{i,t - 1}} &=& \Delta {\rm{LA}}{{\rm{Z}}_t} = {\beta _0} + {\beta _1}{X_i}\nonumber\\
&&+\, {\beta _2}{C_{i1}} + \ldots + {\beta _{j + 1}}{C_{ij}} + {\varepsilon _i}
\end{eqnarray*}$$
3. Attained size model: LAZ at 1 time point (birth, or the end of each interval), LAZ_t_, was regressed on the exposure of interest, X, and a set of covariates, C_1_, …, C_j_, without conditioning on any measurement of LAZ at a previous time point:
  (4) $$\begin{eqnarray*}
{\rm{LA}}{{\rm{Z}}_{it}} = {\beta _0} + {\rm{ }}{\beta _1}{X_i} + {\rm{ }}{\beta _2}{C_{i1}} + \ldots + {\beta _{j + 1}}{C_{ij}} + {\varepsilon _i}
\end{eqnarray*}$$
4. Stunting model: Each child's stunting status (defined as stunted if LAZ < −2 and not stunted if LAZ ≥ −2) was regressed on specific risk factors of interest and relevant covariates using a modified Poisson regression with robust SE variance to obtain an RR (25):
  (5) $$\begin{eqnarray*}
\hskip-5pt{\rm{Pr}}\left[ {{\rm{LA}}{{\rm{Z}}_t} < - 2} \right] = exp({\beta _0} + {\beta _1}X + {\beta _2}{C_1} + \ldots + {\beta _{j + 1}}{C_j})
\end{eqnarray*}$$

We also estimated associations of feeding variables with attained size and stunting at birth to document the potential bias due to reverse causality in estimating the effect of feeding on growth. As a post hoc sensitivity analysis, we also assessed the association of breastfeeding pattern from 0 to 3 mo of age with infant growth from birth to 12 mo of age using a mixed effects model with linear splines (**Supplemental Methods 3**). Multivariable-adjusted associations were expressed as mean differences or RRs and 95% CI.

Sensitivity analyses were conducted using various alternate derivations of the feeding variables using the conditional growth modeling approach (Supplemental Methods 2). Using the conditional growth approach, we also assessed the unadjusted associations of various factors that are not modifiable or not immediately modifiable in the postnatal period (described collectively as “nonmodifiable”): infant sex, birth weight and WLZ at birth, location and mode of delivery, neonatal hospitalization, maternal or paternal education levels and occupations, number of siblings, wealth index, maternal age, maternal 12-mo postpartum BMI, and maternal height.

Comparisons were 2-sided and considered statistically significant if *P* < 0.05. Analyses were complete case without any imputation of missing data and no corrections were made for multiple testing. All analyses were conducted using Stata 15.1 (26).

## Results

### Characteristics of participants and population-average LAZ trajectory

**FIGURE 1:**
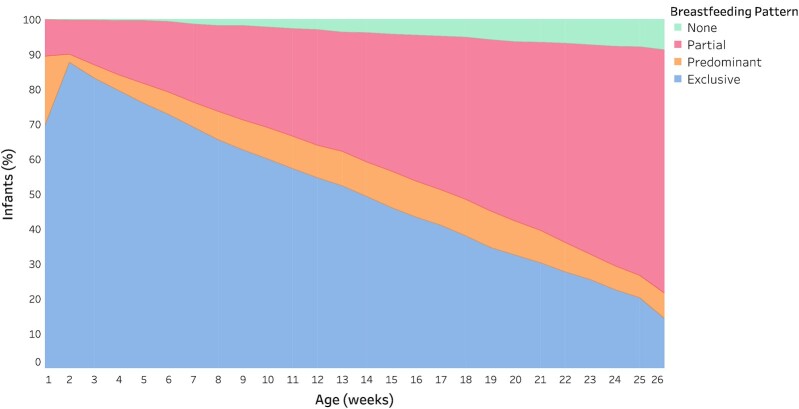
Cumulative infant feeding patterns according to WHO classifications up to 6 mo of age in a birth cohort in Dhaka, Bangladesh (*n* = 1157). For any given week, the breastfeeding pattern for infants was based on the least optimal breastfeeding pattern from the second week up until that week. The proportion of infants exclusively breastfed was relatively low in the first week due to the common practice of prelacteal feeds, so the first week was not included in the derivation of the cumulative breastfeeding pattern classification from week 2 to week 26 shown here and used in the primary analyses. Beyond 1 wk of age, cessation of EBF was defined as any non-EBF exposure. The number of infants contributing breastfeeding data in a given week ranged from 1039 to 1155. EBF, exclusive breastfeeding.

Maternal, household, delivery, and infant characteristics are summarized in **Table 1**, and infant feeding patterns are summarized in **Table 2**. A total of 1157 infants were included in ≥1 age interval–specific analysis (**Supplemental Figure 2**). EBF was common in the newborn period; for example, 87% (*n = *934) of infants were exclusively breastfed at 2 wk of age, although this included 235 infants exposed to prelacteal feeds in the first week of life (**Figure 1**). By 3 mo, 50% of infants (*n = *574) were exclusively breastfed (Table 2) including 117 infants exposed to prelacteal feeds in the first week of life. Using the primary breastfeeding pattern definitions based on weekly recall throughout the 0–6-mo period, only 159 (14%) of the infants included in the analysis met the study criteria for EBF from birth until 6 mo (Figure 1; Table 2).

**TABLE 1:** Infant, maternal, and household characteristics of participants in a birth cohort in Dhaka, Bangladesh

Characteristic	Infants contributing data to ≥1 analysis (*n = *1157)^1^	Infants contributing data to the analysis with the smallest sample size^1^^,^^2^ (*n = *920)
Maternal age, y, median (25th, 75th percentiles)	23 (20, 26)	23 (20, 26)
Maternal 12-mo postnatal BMI, kg/m^2^, mean ± SD	23.7 ± 4.2	23.8 ± 4.2
Maternal height, cm, mean ± SD	150.9 ± 5.4	150.9 ± 5.4
Male infant, *n* (%)	587 (51)	472 (51)
Newborn weight-for-length *z*-score, mean ± SD^3^	−0.8 ± 1.0	−0.8 ± 1.0
Newborn weight-for-gestational-age *z*-score, mean ± SD^4^	−1.2 ± 0.9	−1.2 ± 0.9
Newborn length-for-gestational-age *z*-score, mean ± SD^5^	−0.95 ± 1.02	−0.91 ± 0.97
Gestational age at birth, d, median (25th, 75th percentiles)	274 (267, 280)	274 (268, 281)
Vaginal delivery, *n* (%)	551 (48)	427 (46)
Hospital delivery, *n* (%)	978 (85)	788 (86)
Prelacteal feeds, *n* (%)^6^	227 (26)	184 (25)
Neonatal hospitalization, *n* (%)	169 (15)	134 (15)
Wealth index, mean ± SD	0.0 ± 1.7	0.1 ± 1.6
Maternal occupation: homemaker, *n* (%)	1081 (93)	856 (93)
Paternal occupation, *n* (%)		
Day laborer, rickshaw driver, agricultural worker	121 (10)	96 (10)
Salaried worker	630 (55)	495 (54)
Private business owner, professional	342 (30)	271 (30)
Jobless	20 (1.7)	18 (2)
Other	42 (3.6)	38 (4.1)
Maternal education, *n* (%)		
No schooling	50 (4.3)	43 (4.7)
Incomplete primary schooling or madrasah	245 (21)	188 (20)
Completed primary school	599 (52)	484 (53)
Completed high school	242 (21)	193 (21)
University	21 (1.8)	12 (1.3)
Paternal education, *n* (%)		
No schooling	75 (6.5)	63 (6.8)
Incomplete primary schooling or madrasah	189 (16)	145 (16)
Completed primary school	548 (47)	440 (48)
Completed high school	198 (17)	162 (18)
University	61 (5.3)	48 (5.2)
Unknown	86 (7.4)	62 (6.7)
Number of siblings, median (25th, 75th percentiles)	1 (0, 1)	1 (0, 1)
Age of youngest sibling, *n* (%)		
No other siblings	541 (47)	420 (46)
0–24 mo	34 (2.9)	27 (2.9)
24–36 mo	73 (6.3)	59 (6.4)
37–48 mo	94 (8.1)	74 (8.1)
>48 mo	413 (36)	338 (37)
Any smoking in household, *n* (%)	400 (35)	313 (34)
Home water is treated (boiled, bleached, or filtered), *n* (%)	529 (46)	430 (47)
Observed soap or detergent at wash station in home, *n* (%)	975 (84)	772 (84)

1 Sample size *n = *1157 or *n = *920 in each column respectively, except the following: prelacteal feeds *n = *881 and *n = *732; paternal occupation *n = *1155 and *n = *918; age of youngest sibling *n = *1155 and *n = *918; any smoking in household *n = *1154 and *n = *918.

2 The smallest sample size was for the model in which length-for-age *z*-score from 3 to 6 mo was the outcome and breastfeeding pattern from 0 to 6 mo was the primary exposure.

3 Weight-for-length data for 1059 infants contributing data to ≥1 analysis and 920 infants in models with the smallest sample size.

4 Weight-for-gestational age *z*-scores data for 1097 infants contributing data to ≥1 analysis and 920 infants contributing data to the model with the smallest sample size.

5 Length-for-gestational age z-scores data for 1098 infants contributing data to ≥1 analysis and 920 infants contributing data to the model with the smallest sample size.

6 Infants were classified as having been exposed to prelacteal feeds if they were not exclusively breastfed in the first week of life, but subsequently exclusively breastfed in the second week. The denominator comprises infants who were classified as exclusively breastfed in the second week.

**TABLE 2:** Infant feeding-related factors in the 0–3-mo and 0–6-mo age intervals applied to the analyses of a birth cohort in Dhaka, Bangladesh (*n* = 1157)^1^

	Age interval in which exposure was ascertained	
Infant feeding factor	0–3 mo	0–6 mo
*n* Max	1157	1157
Breastfeeding pattern, *n* (%)		
EBF	574 (50)	159 (14)
Predominant breastfeeding	111 (9.6)	81 (7)
Partial breastfeeding	382 (33)	776 (67)
None (i.e., no breastfeeding)	42 (3.6)	98 (8.5)
Unable to classify	48 (4.1)	43 (3.7)
EBF duration since birth, wk, median (25th 75th percentiles)	13 (5, 13)	13 (5, 22)
Animal source food intake, *n* (%)		
Ever	107 (9.6)	360 (32)
Infant formula intake, *n* (%)		
Ever	394 (35)	704 (62)
Maternal concern about feeding or weight gain, *n* (%)		
Ever	426 (37)	647 (56)
Maternal concern about feeding or weight gain within 1 mo prior to cessation of EBF or end of age interval, *n* (%)		
Yes	223 (20)	277 (25)
No	895 (80)	844 (75)

1 Restricted to infants included in ≥1 analysis assessing the association of infant size with feeding and/or household characteristics. Sample size was 1157 for all variables shown except for animal source food intake (*n = *1111 from 0 to 3 mo and *n = *1116 from 0 to 6 mo); infant formula intake (*n = *1118 from 0 to 3 mo and *n = *1131 from 0 to 6 mo); maternal concern about feeding or weight gain (*n = *1150 from 0 to 3 mo and *n *= 1156 from 0 to 6 mo); and maternal concern in the month prior to cessation of EBF (*n = *1118 from 0 to 3 mo and *n = *1156 from 0 to 6 mo). Details on derivation of feeding-related factors can be found in Supplemental Methods 2. EBF, exclusive breastfeeding.

Mean ± SD newborn LAZ, WAZ, and WFL were −0.95 ± 1.02, −1.20 ± 0.90, and −0.80 ± 1.00, respectively (Table 1). Mean LAZ fluctuated during the 12-mo period; however, SDs remained relatively stable and there was no substantial skewness or asymmetry at any age. Mean LAZ ± SD was −0.87 ± 0.93 at 3 mo, −0.84 ± 0.98 at 6 mo, and −1.00 ± 1.04 at 12 mo (**Supplemental Table 1**; **Supplemental Figure 3**). The observed mean LAZ was always well below that of the theoretical mean (LAZ = 0) that would be expected under optimal conditions for growth (Supplemental Figure 3).

### Feeding patterns and infant growth

Partial and not breastfeeding from 0 to 3 mo were associated with slower conditional growth compared with EBF in the 0–3-mo interval (**Table 3**). However, all groups were similar by 12 mo; specifically, neither partial nor not breastfeeding in the 0–3-mo period was associated with overall growth from 0 to 12 mo (Table 3). Feeding patterns classified on the basis of the 0–6-mo period were not associated with growth from 0 to 6 mo or total growth in the 0–12-mo interval (Table 3). Duration of EBF was not significantly associated with cLAZ in any interval (Table 3). Infants given formula in the 0–3-mo interval had slower average growth in that interval (Table 3); however, those infants experienced relatively faster growth in the 3–6-mo period such that no associations with formula intake were sustained over the 0–6-mo period or to 12 mo (Table 3). Animal-source food intake was not associated with cLAZ in any interval (Table 3). Inferences in all intervals were unchanged after adjusting for maternal concern about infant feeding or growth in the month preceding a change in feeding pattern (**Supplemental Table 2**).

**TABLE 3:** Multivariable-adjusted associations of early postnatal feeding-related factors with linear growth in 5 age intervals of infancy in a birth cohort in Dhaka, Bangladesh, using a residuals model approach^1^

	Age interval in which linear growth outcome was ascertained^2^														
Feeding-related factor (age interval in which exposure was ascertained)	0–3 mo			3–6 mo			0–6 mo			6–12 mo			0–12 mo		
	*n*	Difference in LAZ (95% CI)	*P*	*n*	Difference in LAZ (95% CI)	*P*	*n*	Difference in LAZ (95% CI)	*P*	*n*	Difference in LAZ (95% CI)	*P*	*n*	Difference in LAZ (95% CI)	*P*
Breastfeeding pattern (0–3 mo)	924			922			930			944			977		
EBF	482	ref	ref	474	ref	ref	482	ref	ref	488	ref	ref	510	ref	ref
Predominant	98	0.02 (−0.11, 0.16)	0.742	98	−0.01 (−0.12, 0.10)	0.866	96	0.01 (−0.15, 0.16)	0.946	98	−0.06 (−0.17, 0.05)	0.289	101	−0.06 (−0.23, 0.12)	0.523
Partial	315	−0.11 (−0.20, –0.02)	0.021	319	0.06 (−0.01, 0.13)	0.111	318	−0.01 (−0.11, 0.09)	0.851	322	0.01 (−0.06, 0.08)	0.796	330	0.004 (−0.11, 0.12)	0.938
None	29	−0.30 (−0.54, –0.07)	0.012	31	−0.02 (−0.21, 0.16)	0.831	34	−0.23 (−0.49, 0.02)	0.069	36	0.08 (−0.10, 0.25)	0.405	36	−0.11 (−0.39, 0.17)	0.443
Breastfeeding pattern (0–6 mo)				920			936			950			976		
EBF	—	—	—	130	ref	ref	133	ref	ref	135	ref	ref	138	ref	ref
Predominant	—	—	—	72	−0.05 (−0.19, 0.10)	0.529	69	−0.06 (−0.27, 0.15)	0.568	73	0.04 (−0.11, 0.19)	0.594	75	−0.01 (−0.24, 0.23)	0.966
Partial	—	—	—	643	0.04 (−0.05, 0.14)	0.382	654	0.02 (−0.12, 0.15)	0.789	659	0.07 (−0.03, 0.16)	0.186	680	0.07 (−0.08, 0.22)	0.362
None	—	—	—	75	0.03 (−0.12, 0.17)	0.728	80	−0.07 (−0.27, 0.14)	0.511	83	0.15 (0.004, 0.29)	0.044	83	0.06 (−0.16, 0.29)	0.587
EBF duration from birth, per 1 mo (0–3 mo)	938	0.03 (−0.005, 0.07)	0.086	938	−0.02 (−0.05, 0.02)	0.319	954	0.002 (−0.04, 0.04)	0.943	967	−0.01 (−0.04, 0.02)	0.524	1010	−0.01 (−0.06, 0.04)	0.666
EBF duration from birth, per 1 mo (0–6 mo)	—	—	—	938	−0.01 (−0.03, 0.01)	0.227	954	−0.0004 (−0.02, 0.02)	0.971	967	−0.01 (−0.02, 0.01)	0.467	1010	−0.01 (−0.03, 0.02)	0.590
Animal-source food intake (0–3 mo)	933			933			944			957			994		
Never	845	ref	ref	844	ref	ref	854	ref	ref	866	ref	ref	901	ref	ref
Ever	88	−0.13 (−0.27, 0.01)	0.065	89	0.0003 (−0.11, 0.11)	0.995	90	−0.11 (−0.27, 0.04)	0.158	91	0.06 (−0.06, 0.17)	0.323	93	−0.06 (−0.23, 0.12)	0.517
Animal-source food intake (0–6 mo)				929			942			955			983		
Never	—	—	—	637	ref	ref	642	ref	ref	650	ref	ref	669	ref	ref
Ever	—	—	—	292	0.03 (−0.04, 0.10)	0.434	300	−0.01 (−0.11, 0.09)	0.818	305	0.004 (−0.07, 0.08)	0.902	314	−0.02 (−0.13, 0.08)	0.657
Formula intake (0–3 mo)	934			935			947			960			998		
Never	615	ref	ref	608	ref	ref	620	ref	ref	627	ref	ref	655	ref	ref
Ever	319	−0.13 (−0.22, −0.04)	0.003	327	0.08 (0.01, 0.15)	0.028	327	−0.02 (−0.12, 0.08)	0.697	333	0.02 (−0.05, 0.09)	0.595	343	0.01 (−0.10, 0.12)	0.825
Formula intake (0–6 mo)				933			947			961			992		
Never	—	—	—	362	ref	ref	364	ref	ref	373	ref	ref	382	ref	ref
Ever	—	—	—	571	0.05 (−0.02, 0.11)	0.171	583	0.01 (−0.09, 0.10)	0.915	588	0.09 (0.02, 0.16)	0.012	610	0.09 (−0.01, 0.20)	0.089

1 All models adjusted for the following covariates: assigned treatment group in the MDIG trial, maternal height, neonatal illness (ever/never), delivery location, delivery type, maternal and paternal education, wealth index, number of children, maternal postnatal BMI, maternal age, infant sex, newborn weight for length *z*-score, and gestational age at birth. EBF, exclusive breastfeeding; LAZ, length-for-age *z*-score; MDIG, Maternal Vitamin D and Infant Growth trial.

2 Median (IQR) duration of interval in days: 0–3 mo: 90 (90–91); 3–6 mo: 91 (91–91); 0–6 mo: 181 (181–182); 6–12 mo: 182 (182–183); 0–12 mo: 363 (363–365).

### Household characteristics and infant growth

In primary models (residuals method), not having soap at the wash station was associated with faster conditional growth in the 6–12-mo interval but the association was attenuated and nonsignificant for the complete birth to 12-mo period (**Table 4**). Reported household smoking and water treatment were not associated with growth in any interval (Table 4). Inferences were unchanged when household data collected at 9 mo postpartum were used instead of characteristics reported in the prenatal period (**Supplemental Table 3**). Vitamin D supplementation was not associated with growth in any model (data not shown).

**TABLE 4:** Multivariable-adjusted associations of household factors with linear growth in 5 age intervals of infancy in a birth cohort in Dhaka, Bangladesh, using a residuals model approach^1^

	Age interval in which linear growth outcome was ascertained^2^														
Household-related factor	0–3 mo			3–6 mo			0–6 mo			6–12 mo			0–12 mo		
	*n*	Difference in LAZ (95% CI)	*P*	*n*	Difference in LAZ (95% CI)	*P*	*n*	Difference in LAZ (95% CI)	*P*	*n*	Difference in LAZ (95% CI)	*P*	*n*	Difference in LAZ (95% CI)	*P*
Smoking in household	1007			1054			1018			1070			1069		
Never	665	ref	ref	687	ref	ref	668	ref	ref	696	ref	ref	697	ref	ref
Ever	342	0.03 (−0.05, 0.12)	0.441	367	−0.04 (−0.11, 0.03)	0.243	350	−0.03 (−0.13, 0.07)	0.547	374	−0.02 (−0.09, 0.05)	0.529	372	−0.03 (−0.13, 0.08)	0.637
Water treatment	1010			1057			1021			1073			1072		
Untreated	552	ref	ref	576	ref	ref	557	ref	ref	585	ref	ref	580	ref	ref
Treated	458	−0.08 (−0.16, 0.01)	0.075	481	0.03 (−0.04, 0.09)	0.379	464	−0.05 (−0.14, 0.05)	0.312	488	0.04 (−0.03, 0.11)	0.266	492	0.02 (−0.08, 0.13)	0.680
Observed soap	1010			1057			1021			1073			1072		
Soap or detergent	848	ref	ref	889	ref	ref	854	ref	ref	903	ref	ref	902	ref	ref
No soap	162	0.01 (−0.11, 0.13)	0.862	168	−0.01 (−0.10, 0.07)	0.766	167	−0.02 (−0.15, 0.11)	0.735	170	0.12 (0.03, 0.21)	0.012	170	0.08 (−0.06, 0.23)	0.252

1 All models adjusted for the following covariates: assigned treatment group in the MDIG trial, maternal height, neonatal illness (ever/never), delivery location, delivery type, maternal and paternal education, wealth index, number of children, maternal postnatal BMI, maternal age, infant sex, newborn weight for length *z*-score, and gestational age at birth. LAZ, length-for-age *z*-score; MDIG, Maternal Vitamin D and Infant Growth trial.

2 Median (IQR) duration of interval in days: 0–3 mo: 90 (90–91); 3–6 mo: 91 (91–91); 0–6 mo: 181 (181–182); 6–12 mo: 182 (182–183); 0–12 mo: 363 (363–365).

### Alternative growth modeling approaches

For most associations, estimates and inferences obtained from the 2 other interval-growth approaches, ANCOVA (**Supplemental Table 4**; **Table 5**) and change-score models (Table 5; **Supplemental Table 5**), were similar to those from the conditional growth models. None of the modeling approaches suggested sustained associations of any of the early-life exposures with growth up to 12 mo. Considering those associations examined using interval growth approaches, there were 14/69 (19%) exposure-outcome associations for which the inference from ≥1 modeling approach was statistically significant. For 4 of these 14 (14%) associations, inferences differed across any 2 of the interval growth approaches, although differences in point estimates were minor (**Supplemental Table 6**). For these 4 associations with discrepancies, the change-score model differed from the residuals approach (primary approach). For example, water treatment in the home was associated with a statistically significant 0.11 lower LAZ from 0 to 3 mo of age in the change-score model, but the effect was attenuated and nonsignificant in the other interval growth approaches, and was essentially null in the attained size model (Supplemental Table 6). Conversely, the ANCOVA model did not differ from the residuals approach, and the point estimates and CIs were essentially identical (Supplemental Table 6).

**TABLE 5:** Comparison of 5 alternative statistical modeling approaches to estimate multivariable-adjusted associations of infant breastfeeding pattern from 0 to 3 mo of age with linear growth or attained stature in 3 age intervals of infancy in a birth cohort in Dhaka, Bangladesh^1^

	Age interval in which outcome (linear growth or attained stature) was ascertained^2^								
	0–3 mo			0–6 mo			0–12 mo		
Breastfeeding pattern (0–3 mo)	*n*	Difference in LAZ or RR (95% CI)^3^	*P*	*n*	Difference in LAZ or RR (95% CI)^3^	*P*	*n*	Difference in LAZ or RR (95% CI)^3^	*P*
EBF	482	ref	ref	482	ref	ref	510	ref	ref
Predominant BF									
Residuals	98	0.02 (−0.11, 0.16)	0.742	96	0.01 (−0.15, 0.16)	0.946	101	−0.06 (−0.23, 0.12)	0.523
ANCOVA	98	0.02 (−0.12, 0.16)	0.776	96	0.0002 (−0.16, 0.16)	0.997	101	−0.06 (−0.24, 0.11)	0.486
“Change score”	98	0.06 (−0.09, 0.22)	0.412	96	0.05 (−0.13, 0.23)	0.570	101	−0.02 (−0.22, 0.18)	0.859
Attained size	98	−0.06 (−0.24, 0.13)	0.552	96	−0.07 (−0.26, 0.13)	0.494	101	−0.10 (−0.31, 0.10)	0.305
Partial BF									
Residuals	315	−0.11 (−0.20, −0.02)	0.021	318	−0.01 (−0.11, 0.09)	0.851	331	0.004 (−0.11, 0.12)	0.938
ANCOVA	315	−0.11 (−0.20, −0.02)	0.019	318	−0.01 (−0.12, 0.09)	0.829	331	0.001 (−0.11, 0.12)	0.986
“Change score”	315	−0.09 (−0.19, 0.01)	0.091	318	0.004 (−0.12, 0.12)	0.950	331	0.03 (−0.10, 0.16)	0.645
Attained size	315	−0.14 (−0.26, −0.02)	0.025	318	−0.03 (−0.16, 0.10)	0.632	331	−0.03 (−0.16, 0.10)	0.668
None (no BF)									
Residuals	29	−0.30 (−0.54, −0.07)	0.012	34	−0.23 (−0.49, 0.02)	0.069	36	−0.11 (−0.39, 0.17)	0.443
ANCOVA	29	−0.30 (−0.54, −0.07)	0.011	34	−0.24 (−0.49, 0.01)	0.062	36	−0.12 (−0.39, 0.16)	0.409
“Change score”	29	−0.26 (−0.53, 0.01)	0.055	34	−0.18 (−0.47, 0.11)	0.228	36	−0.05 (−0.37, 0.27)	0.774
Attained size	29	−0.37 (−0.69, −0.05)	0.023	34	−0.32 (−0.64, −0.01)	0.044	36	−0.19 (−0.51, 0.13)	0.255
Stunting model	924	Estimates below are RRs (95% CI)		930	Estimates below are RRs (95% CI)		977	Estimates below are RRs (95% CI)	
EBF	482	ref	ref	482	ref	ref	510	ref	ref
Predominant	98	1.40 (0.76, 2.57)	0.283	96	1.03 (0.56, 1.91)	0.927	101	1.05 (0.65, 1.70)	0.840
Partial	315	2.02 (1.31, 3.12)	0.001	318	1.32 (0.86, 2.02)	0.210	330	0.98 (0.69, 1.37)	0.886
None	29	2.36 (0.80, 6.95)	0.119	34	2.15 (0.91, 5.08)	0.080	36	1.35 (0.61, 2.98)	0.457

1 All models adjusted for the following covariates: assigned treatment group in the MDIG trial, maternal height, maternal and paternal occupation, maternal and paternal education, asset index, neonatal illness, delivery location, delivery type, maternal and paternal education, wealth index, number of children, maternal postnatal BMI, maternal age, infant sex, birth weight, and birth weight for length. See text for explanation of the statistical models. BF, breastfeeding; EBF, exclusive breastfeeding; LAZ, length-for-age *z*-score; MDIG, Maternal Vitamin D and Infant Growth trial.

2 Median (IQR) duration of interval in days: 0–3 mo: 90 (90–91); 0–6 mo: 181 (181–182); 0–12 mo: 363 (363–365).

3 Effect estimates have different interpretations in each modeling approach, but are referred to generically as differences in LAZ (across feeding groups) for the residuals, ANCOVA, change score, and attained size models. Stunting model is a logistic regression model that yields ORs.

Attained size (Table 5; **Supplemental Table 7**) and stunting models (Table 5; **Supplemental Table 8**) yielded inferences that were generally consistent with the interval growth models. The attained size model differed from the residuals approach for 4 of 9 associations for which both analyses were performed and where ≥1 of the approaches yielded a significant inference (Supplemental Table 6). One notable difference was that animal-sourced food intake (0–3 mo) was associated with smaller attained LAZ and higher risk of stunting at 6 mo of age, whereas the effects were smaller and nonsignificant for all 3 of the interval growth modeling approaches (Supplemental Table 6). The attained size and stunting models also provided evidence of reverse causality or other unmeasured confounding: LAZ at birth was lower in those who had received animal-sourced foods from 0 to 3 mo (Supplemental Table 7); also, not breastfeeding (compared with EBF) from 0 to 3 mo was associated with higher rates of stunting at birth, and every additional month of EBF was associated with lower rates of stunting at birth (Supplemental Table 8).

A post hoc multilevel mixed effects linear model to assess the modifying effect of feeding pattern from 0 to 3 mo on the LAZ-by-age trajectory yielded the same inferences as the primary residuals approach; specifically, partial breastfeeding (compared with EBF) was associated with slower growth from 0 to 3 mo, but correspondingly faster growth from 3 to 6 mo, and not associated with growth from 6 to 12 mo (**Supplemental Table 9**). As with all the other modeling approaches, we did not observe sustained associations of breastfeeding pattern from 0 to 3 mo with infant growth to 12 mo of age (Supplemental Table 9).

### Sensitivity analyses

In contrast to primary analyses, there were no significant associations between feeding pattern and growth in any interval when a strict breastfeeding definition was used (Alternative A), allowing for a single deviation in any week (Alternative D), when only including infants with no missing breastfeeding data (Alternative F), or when the breastfeeding classification was based only on data collected 24 h prior to measurement (Alternative G) (**Supplemental Table 10**). As well, when allowing infants to be classified with a nonexclusive breastfeeding pattern (rather than “unable to classify”) even if they had missing feeding data in the 2 wk prior to measurement (Alternative C), or when the breastfeeding classification was based only on the 2 wk of data collected prior to measurement (Alternative E), not breastfeeding was associated with relatively faster growth in the 6–12-mo time period compared with EBF (Supplemental Table 10). Also, in contrast to the primary analyses, when stricter age ranges were used to group the growth measurements, not breastfeeding was not associated with slower growth over any time period (data not shown). All other sensitivity analyses showed no changes in inferences.

### Risk factors that are nonmodifiable or not amenable to intervention in the immediate postnatal period

Girls had 0.17 (95% CI: 0.09, 0.25), 0.13 (95% CI: 0.04, 0.23), and 0.17 (95% CI: 0.06, 0.27) greater cLAZ in the 0–3-, 0–6-, and 0–12-mo intervals, respectively, compared with boys. Higher maternal BMI was associated with slower growth from 0 to 3 mo, and faster growth from 6 to 12 mo (**Supplemental Table 11**). Maternal height was positively associated with growth in all intervals. Higher levels of both maternal and paternal education were associated with faster growth in the 0–6-, 6–12-, and 0–12-mo intervals, with infants of parents with completed secondary education or higher growing fastest (Supplemental Table 11). Compared with having no siblings, having ≥2 siblings in the household was associated with slower growth in the 0–3-, 0–6-, and 0–12-mo periods.

## Discussion

Feeding patterns or selected modifiable household characteristics did not have sustained associations with linear growth from birth to 12 mo in Bangladeshi infants. Overall, findings were similar using alternative statistical models, although minor differences in some estimates and inferences underscored the potential impact of model choice on studies of child growth (16). The average growth trajectory was below the theoretical optimal (mean LAZ = 0), and the entire distribution was shifted down, indicating that nearly the entire cohort experienced growth faltering compared with a healthy population. This highlights that studies of between-child variability in growth are inadequate for understanding the community factors that cause whole-population faltering (27).

Partial or no breastfeeding (compared with EBF), and formula intake, were associated with relatively slower growth from 0 to 3 mo of age, but there were no sustained effects to 12 mo of age. EBF from 0 to 6 mo (recommended by the WHO) was not associated with faster growth (or lower risk of stunting) at 12 mo compared with predominant or partial breastfeeding. Although upstream social determinants of child growth are widely acknowledged (1), global health responses to child undernutrition have tended to assume that “stunting is an outcome of maternal undernutrition and inadequate infant and young child feeding” (28). EBF to 6 mo is often advocated to reduce stunting (29, 30) despite evidence from LMICs that early infant feeding patterns are unrelated to linear growth (4, 31–34), and that EBF promotion does not improve growth (14, 35) or might increase the risk of stunting (36–38) even though it protects against diarrhea (39). In the PROBIT trial in Belarus, breastfeeding promotion led to greater length increases in the first 3 mo, but there was no effect by 12 mo of age (40), mirroring the present findings. In high-income countries, formula-fed infants can have greater weight gain (41), particularly beyond 3–4 mo (42, 43), but length has not been consistently associated with feeding (41). The present findings contribute further insights into age window–specific feeding-growth associations in an LMIC: infants who are exclusively/predominantly breastfed from 0 to 3 mo might grow slightly faster up to 3 mo, but without sustained effects. Moreover, observed associations of early feeding patterns with length at birth suggest residual confounding of the feeding-growth associations. “Maternal concern” about a baby's growth or feeding did not explain any observed associations, but it is difficult to disentangle a baby's size/growth from the caregiver's decisions about feeding (44, 45). Overall, the evidence indicates that although EBF promotion is strongly justified by other benefits, it should not be expected to improve linear growth or reduce the prevalence of stunting in LMICs.

Other modifiable household resources and behaviors were not the primary focus of the study and were limited by heterogeneity in some measures of facilities and practices at the household level. However, the lack of beneficial associations of water treatment practices or soap at the wash station broadly agreed with the null effects of water sanitation and hygiene (WaSH) interventions on linear growth in recent trials in Kenya, Zimbabwe, and Bangladesh (46). WaSH appears to be a key example of a class of early-life exposures for which micro-level household or behavioral modifications could be ineffective because they do not fundamentally change the community-level causes of widespread factors that constrain growth, such as microbial contamination (46). Furthermore, other studies have similarly shown the dominant influence of upstream biological factors (e.g., maternal stature) on early childhood height (47), emphasizing the limited role of readily modified individual-level factors in the postnatal period.

In contrast to child growth studies that apply a single modeling approach (15), we considered if conclusions differed depending on the approach to estimating exposure-growth associations. Most of the null findings were similar across models, but several associations were only statistically significant by certain models. We acknowledge the limitations of cross-model comparisons based on a *P* value threshold-driven hypothesis testing framework (i.e., point estimates were generally similar even if precision differed); yet, in some circumstances model choice can influence estimates and could therefore account for discrepancies between otherwise similar studies. In a previous study, the change-score model was preferred over ANCOVA because it avoids overadjustment for covariates that temporally precede and are associated with the baseline size parameter in ANCOVA models (16); however, the authors of that study did not apply the residuals method, which can overcome this limitation and has been widely adopted elsewhere (48, 49). The residuals and ANCOVA models can sometimes yield different results (50), but we found they produced similar estimates. Residuals and ANCOVA models uniquely account for regression to the mean—the expectation that infants who were smaller or larger than peers at the beginning of an interval will tend to exhibit “catch-up” or faltering (51, 52). As demonstrated in studies in which interval growth was the exposure rather than outcome (53), the models are algebraically related to one another, and yield regression coefficients with slightly different but complementary interpretations. Using a more flexible longitudinal (repeated-measures) model can offer statistical advantages and is particularly useful when there is variability in the timing of anthropometric measurements (54). However, in a sensitivity analysis using mixed-effects regression with linear splines, we found that inferences were similar to the simpler approaches, likely because the timing of anthropometric measurements in this cohort was relatively uniform. Integration of findings from interval-growth and attained size models clarified the specific periods of growth that gave rise to observed differences in average LAZ at any given age; for example, differences between no breastfeeding and EBF at 3 mo were largely attributable to slower postnatal growth from 0 to 3 mo in the no-breastfeeding group. Exposure definitions (e.g., breastfeeding pattern) influenced the magnitude and direction of associations to an even greater extent than statistical model choice, although inferences were generally unchanged.

This was an observational analysis of a randomized trial dataset and was therefore limited to exposure variables for which data were available, coherently characterized, and had sufficient between-infant heterogeneity in the study population. As in all observational studies, estimates likely remained biased by unmeasured confounding. Estimating the relation between a time-varying exposure (feeding) and time-varying outcome (length) is challenging, and the findings might have differed if we had more frequent anthropometric assessments or had used other methods to address treatment-confounder feedback. In addition, because the primary feeding variables were based on maternal/caregiver report, we cannot exclude an effect of response bias (e.g., social desirability bias such that mothers might have tended to underreport feeding practices that are inconsistent with public health recommendations). Another limitation was the absence of a marked decline in LAZ previously observed in Dhaka and other LMICs (11), yet this cohort was still substantially shorter than normal, indicating suboptimal conditions for growth. Strengths of the study were the high quality of anthropometric data and prospective ascertainment of feeding status in the first 6 mo. We used multiple approaches to examine concurrent and lagged effects of feeding in defined age windows, and numerous sensitivity analyses. Expected robust associations of size/growth with nonmodifiable factors (e.g., parental education) suggested that null findings for modifiable exposures were unlikely to be due to imprecision in the anthropometric outcome measurements.

In conclusion, early infant feeding patterns or other selected modifiable household characteristics did not explain between-child variations in infant linear growth in a low-income setting where small size at birth and postnatal linear growth faltering are pervasive. Choice of the statistical growth model, which gave rise to minor differences in some estimates, should be considered in future studies. As observed in LMICs in general, the LAZ distribution and population-average pattern of linear growth suggest that the prenatal and postnatal exposures that constrain linear growth are mainly upstream social phenomena or ubiquitous factors (27). Programs and policies to promote healthy infant growth in LMICs should target structural causes of undernutrition rather than blame maternal or household caregiving practices and behaviors.

## Supplementary Material

nzab077_Supplemental_File

## Data Availability

Data described in the manuscript, code book, and analytic code will be made available upon request pending application and approval.

## Notes

Funding for the MDIG trial including all data collection as well as data analyses for this MDIG substudy was provided by the Bill & Melinda Gates Foundation; however, the authors did not receive any external funding that was specifically designated to support the preparation of this manuscript.

Author disclosures: The authors report no conflicts of interest.

Supplemental Methods 1–3, Supplemental Tables 1–11, and Supplemental Figures 1–3 are available from the “Supplementary data” link in the online posting of the article and from the same link in the online table of contents at https://academic.oup.com/cdn/.

Abbreviations used: cLAZ, conditional length-for-age *z*-score; DAG, directed acyclic graph; EBF, exclusive breastfeeding; LMICs, low- and middle-income countries; LAZ, length-for-age *z*-score; MDIG, Maternal Vitamin D and Infant Growth trial; SGA, small for gestational age; WaSH, water, sanitation, and hygiene; WAZ, weight-for-age *z*-score; WFL, weight-for-length *z*-score.
