# Supplementary material for: Do Early Infant Feeding Practices and Modifiable Household Behaviors Contribute to Age-Specific Interindividual Variations in Infant Linear Growth? Evidence from a Birth Cohort in Dhaka, Bangladesh
Source: Curr Dev Nutr. 2021 Apr 30;5(5):nzab077. doi: 10.1093/cdn/nzab077 (PMC8163422; doi:10.1093/cdn/nzab077)
Supplement: nzab077_Supplemental_File [file nzab077_supplemental_file.pdf]

Supplemental Material

Do early infant feeding practices and modifiable household behaviors contribute to age-specific inter-individual variations in infant linear growth? Evidence from a birth cohort in Dhaka, Bangladesh

Sarah L. Silverberg, Huma Qamar, Farhana K. Keya, Shaila S. Shanta, M. Munirul Islam, Tahmeed Ahmed, Joy Shi, Davidson H. Hamer, Stanley Zlotkin, Abdullah Al Mahmud, Daniel E. Roth

Daniel Roth
Hospital for Sick Children
686 Bay Street, Toronto, Canada
daniel.roth@sickkids.ca

Table of Contents

Supplemental Methods 1

Method 1. Description of the MDIG trial Eligibility Criteria 1

Method 2. Definitions and derivation of anthropometry and feeding variables 1

Method 3. Description of post-hoc sensitivity analysis using a mixed effect model 3

Supplemental Table 1. Characteristics of the length-for-age z-score (LAZ) distributions at birth, 3 months, 6 months, and 12 months of age. 4

Supplemental Table 2. Association of breastfeeding pattern from 0-3 months of age or 0-6 months of age with linear growth in two age intervals (0-3 months or 3-6 months of age) with or without adjustment for reported maternal concern about infant feeding or weight gain, using the residuals model approach[1] 5

Supplemental Table 3. Associations of risk factors measured at baseline (2nd trimester of pregnancy) or 9-months postpartum with linear growth at the 6- to 12-month age interval, using a residuals model approach[1] 6

Supplemental Table 4. Multivariable-adjusted associations of early postnatal feeding-related factors and household factors with linear growth in five age intervals of infancy in a birth cohort in Dhaka, Bangladesh, based on the ANCOVA modeling approach[1] 7

Supplemental Table 5. Multivariable-adjusted associations of early postnatal feeding-related factors and household factors with linear growth in five age intervals of infancy in a birth cohort in Dhaka, Bangladesh, based on the ‘change score’ modeling approach[1] 8

Supplemental Table 6. Comparison of effect estimates for the associations of feeding-related and household factors with linear growth outcomes in different age intervals of infancy, using five different growth or size modeling approaches, restricted to associations for which there were significant associations using at least one modeling approach. 9

Supplemental Table 7. Multivariable-adjusted associations of early postnatal feeding-related factors and household factors with linear growth at four ages of infancy in a birth cohort in Dhaka, Bangladesh, using the attained size modeling approach[1] 10

Supplemental Table 8. Multivariable-adjusted associations of early postnatal feeding-related factors and household factors with stunting (length-for-age z-score < -2 SD below the age- and sex-specific median) at four time points[1] 11

Supplemental Table 9. Multivariable association of breastfeeding pattern from 0 to 3 months of age with change in infant length-for-age z-scores in three age intervals and at 12 months of age, using a mixed effects model with linear splines [1,2] 12

Supplemental Table 10. Sensitivity analyses of associations of early postnatal feeding-related factors with linear growth in four age intervals of infancy using alternative breastfeeding classification schemes, exclusive breastfeeding duration criteria, animal intake criteria and formula intake criteria in the first 6 months of life.[1] 13

Supplemental Table 11. Unadjusted associations of non-modifiable risk factors with linear growth at four ages of infancy in a birth cohort in Dhaka, Bangladesh, using the residuals modeling approach[1] 15

Supplemental Figure 1: Conceptual model depicting breastfeeding as exposure of interest related to infant growth. Factors in red are hypothesized confounders... 16

Supplemental Figure 2: Participant eligibility flow diagram. 17

Supplemental Figure 3: Average length-for-age z-score trajectory from birth to twelve months of life. 18

Supplemental References 19

Supplemental Methods

Method 1. Description of the MDIG trial Eligibility Criteria

Pregnant women (n=1300) aged 18 years and above and enrolled at 17-24 weeks gestation were randomized into one of five vitamin D treatment arms: 0 IU/week from prenatal enrolment until delivery and 0 IU/week until 26 weeks; 4200 IU/week prenatal and 0 IU/week postpartum; 16800 IU/week prenatal and 0 IU/week postpartum; 28000 IU/ prenatal and 0 IU/week postpartum; or, 28000 IU/week in both prenatal and postpartum periods.

Method 2. Definitions and derivation of anthropometry and feeding variables

Anthropometry

The birth measurement was the earliest measurement within the first 45 days of life. The 3-month measurement was that which was closest to day 91 (but between day 46 and 136, inclusive). The 6-month measurement was that which was closest to day 182, but between 137 and 227 days. The 12-month measurement was that which was closest to 365, but between 320 and 410. For any infant, if the time between the 0 and 3 month measurements or the 3 and 6 month measurements were fewer than 60 days apart, that infant was excluded from the corresponding analysis where length in that interval was the outcome. Similarly, infants were excluded from analyses where length in the 6 to 12 month interval was the outcome if the 6 and 12 month measurements were fewer than 152 days or more than 212 days apart. If the birth and 12 month measurements were fewer than 304 days apart, the infant would be excluded from analyses where length in the birth to 12 month interval was the outcome.

Risk Factors

Breastfeeding Classification

Each child-week was classified as exclusively breastfed, predominantly breastfed, partially breastfed or not breastfed, per the WHO categories (1). Maternal/caregiver report of the infant's feeding 24 hours/and or 7 days preceding each visit was combined to classify breastfeeding in each week during the first 3 months and the first 6 months of life. In order to reconcile rare discrepancies between the 24-hour and 7-day recall items relating to breast milk intake in the caregiver interview, an infant who was indicated as being breastfed in the last week *or* the last 24-hour period, with no alternative liquids/foods was classified as exclusively breastfed. This combination of the 24-hour and 7-day recall were similarly applied to predominant breastfeeding pattern but with exposures to any additional

liquids and to partial breastfeeding pattern but with exposures to any solid/semi-solid/soft foods. No breastfeeding required the caregiver to indicate no breastmilk was given to the infant in both the 24-hour and 7-day recalls. To aggregate weekly data, for each time period of interest, the assigned breastfeeding category corresponded to the least optimal breastfeeding category among any of the observed child-weeks. If an infant was missing breastfeeding data in the last 2 weeks of observation, the infant was designated ‘not able to be classified’. Due to the high prevalence of pre-lacteal feeds in this population, the primary derivation of this variable ignored the breastfeeding pattern in the first week to classify breastfeeding in the 0-3 and 0-6 month time periods.

In sensitivity analyses, we derived breastfeeding pattern within the first 3 and the first 6 months of life: 1) including feeding data from the first week of life, not allowing for any deviations in any week; 2) requiring the caregiver to indicate that the infant was breastfed in both the 24-hour and 7-day recalls (rather than at least one of the 2 recalls) in order to be classified as exclusively breastfed or predominantly breastfed, and requiring the infant to have been breastfed over at least the 7-day period to be classified as partially breastfed; 3) allowing for a single deviation in any observed child-week but still excluding the first week of life (e.g., if in the first 3 months, a child was partially breastfed in a single week but otherwise exclusively breastfed, then the single week deviation would be ignored and the child would be classified as exclusively breastfed in the first 3 months); 4) allowing for missing data in the last 2 weeks of the classification time period among infants who were not exclusively breastfed; 5) using the last 2 observation weeks (i.e., feeding pattern in weeks 12 and 13 or weeks 25 and 26 to derive feeding in the first 3 and 6 months, respectively); 6) using data from infants who had no missing breastfeeding data in the first 3 or 6 months of life; and 7) using a 24-hour recall period rather than a 7 day period preceding the visit.

Duration of EBF

Duration of exclusive breastfeeding (EBF) in the first 3 and 6 months was the number of weeks since birth that an infant was reported to be exclusively breastfed. In any given week, an infant was identified as having been exclusively breastfed if the caregiver indicated that the infant was breastfed in the last week *or* the last 24-hour period and was not given any alternative liquids or foods. For infants for whom a period of EBF was separated from a period of non-EBF with missing child-weeks of observational data, the duration of EBF was set to be the average between the last known week of EBF and the first week of known non-EBF. For infants for whom a period of exclusive breastfeeding was followed by weeks of missing data with no dietary data available following the missing weeks, the duration of EBF was set to be halfway between the end of the interval and the last known week of EBF. As with breastfeeding pattern, the primary derivation of duration of EBF ignored the first week of life and assumed that the infant was EBF due to the high prevalence of pre-lacteal feeds. Therefore, the minimum duration of EBF was 1 week.

In sensitivity analyses, we used the following alternate derivations of duration of EBF in the first 3 and 6 months: 1) allowing for 1 week of deviation from exclusive breastfeeding, excluding the first week of life; 2) including the first week of life, thereby not allowing for any deviations from EBF in any week; 3) requiring the infant to have been indicated as breastfed by the caregiver in both

the 24-hour and 7-day recalls (rather than at least one of the 2 recalls) in order to be considered exclusively breastfed; 4) using the last known week of EBF as the duration of EBF rather than taking the average between the last known week of EBF and the first week of known EBF or between the last known week of EBF and the end of the age interval; and 5) using the end of the time interval as the duration of EBF for infants who were exclusively breastfed until a certain week followed by missing data up until the end of the interval.

Additional Sensitivity Analyses

The primary derivation of the binary variables (ever/never) for formula exposure and animal source food exposure (i.e., any of milk, yoghurt, cheese, meats and organ meats, eggs, or fish) included only those infants for whom there were available feeding data for at least half of the weekly visits during the respective time period. In sensitivity analyses, we derived formula exposure and animal exposure variables that included infants who completed less than half of the weekly visits. We also generated an additional animal exposure variable for sensitivity analyses whereby infants were classified as having consumed animal products using data from weekly visits (i.e. the primary feeding data source) as well as data on animal product consumption collected from mothers retrospectively during an interview conducted later in infancy.

To address the robustness of the inferences based on variations in anthropometric outcome definitions, the following sensitivity analyses were performed: 1) relaxed the interval duration requirements to permit inclusion of measurements a minimum of 45 days apart rather than 60 days apart; or 2) Increased the stringency of the interval duration requirement by narrowing the timeframe of each measurement, such that the birth measurement was required to be between 0-14 days, the second measurement between 77-105 days, the third measurement between 168-196 days, and the fourth measurement between 351-379. For these sensitivity analyses, we used the primary derivation of breastfeeding pattern as described above.

Method 3. Description of post-hoc sensitivity analysis using a mixed effect model

As a post-hoc sensitivity analysis, we assessed the association of breastfeeding pattern from 0 to 3 months of age with infant growth from birth to 12 months of age using a mixed effects model with linear splines, infant-specific random intercepts and random slopes. Knots were placed at 91 days and 182 days, resulting in 3 spline terms that captured the birth to 3-month interval, 3 to 6 month interval, and 6 to 12-month interval of growth. An interaction between each of the spline terms and breastfeeding pattern was estimated to assess the association of breastfeeding pattern with the change in growth in the interval.

Supplemental Table 1. Characteristics of the length-for-age z-score (LAZ) distributions at birth, 3 months, 6 months, and 12 months of age.

Timing	N	Mean LAZ (SD)	Skewness[2]	Kurtosis[2]	P[1]
Birth [3]	1098	-0.95 (1.02)	-0.09	3.26	0.70
3 months [4]	1094	-0.87 (0.93)	-0.16	3.18	0.54
6 months [5]	1111	-0.84 (0.98)	-0.05	3.08	0.53
12 months [6]	1128	-1.00 (1.04)	-0.07	3.05	0.90

1 p-value for the Kolmogorov- Smirnov test for normality
[2] In a normal distribution, the skewness is expected to be near 0 and kurtosis near 3.
[3] LAZ measurements collected within 45 days of birth
[4] LAZ measurements collected between 62 days and 135 days of age
[5] LAZ measurements collected between 162 days and 223 days of age
[6] LAZ measurements collected between 364 days and 409 days of age

Supplemental Table 2. Association of breastfeeding pattern from 0-3 months of age or 0-6 months of age with linear growth in two age intervals (0-3 months or 3-6 months of age) with or without adjustment for reported maternal concern about infant feeding or weight gain, using the residuals model approach[1]

**Feeding-related factor (age interval in which exposure was ascertained)**	**Age interval in which outcome (linear growth) was ascertained**[2]											
	**0-3 Months**						**3-6 Months**					
	**Without adjustment for presence of any maternal concern during the breastfeeding period**			**With adjustment for presence of any maternal concern during the breastfeeding period**			**Without adjustment for presence of any maternal concern during the breastfeeding period**			**With adjustment for presence of any maternal concern during the breastfeeding period**		
	**N**	Difference in LAZ (95% CI)	**p**	**N**	Difference in LAZ (95% CI)	**p**	**N**	Difference in LAZ (95% CI)	**p**	**N**	Difference in LAZ (95% CI)	**p**
**Breastfeeding pattern (0-3 mo)**	924			924			922			922		
EBF	482	ref	ref	482	ref	ref	474	ref	ref	474	ref	ref
Predominant	98	0.02 (-0.12,0.15)	0.820	98	0.04 (-0.10,0.17)	0.584	98	-0.01 (-0.12,0.10)	0.887	98	-0.02 (-0.13,0.09)	0.742
Partial	315	-0.12 (-0.21,-0.03)	0.011	315	-0.10 (-0.19,-0.01)	0.032	319	0.06 (-0.01,0.13)	0.095	319	0.06 (-0.02,0.13)	0.139
None	29	-0.31 (-0.55,-0.07)	0.010	29	-0.29 (-0.52,-0.05)	0.017	31	-0.02 (-0.20,0.17)	0.855	31	-0.03 (-0.21,0.16)	0.766
**Breastfeeding pattern (0-6 mo)**							920			920		
EBF	–	–	–	–	–	–	130	ref	ref	130	ref	ref
Predominant	–	–	–	–	–	–	72	-0.05 (-0.20,0.09)	0.489	72	-0.04 (-0.19,0.10)	0.572
Partial	–	–	–	–	–	–	643	0.04 (-0.05,0.13)	0.409	643	0.05 (-0.05,0.14)	0.307
None	–	–	–	–	–	–	75	0.02 (-0.12,0.17)	0.737	75	0.04 (-0.10,0.19)	0.582

[1]All models included the following additional covariates: assigned treatment group in the MDIG trial, maternal height, paternal occupation, maternal and paternal education, asset index, neonatal illness, delivery location, delivery mode, maternal and paternal education, wealth index, number of children, maternal postnatal BMI, maternal age, infant sex, newborn weight-for-length z-score, and gestational age at birth.
[2]Median (IQR) duration of interval in days: 0-3 Months: 90 (90-91); 3-6 Months: 91 (91-91); 0-6 Months: 181 (181-182); 6-12 Months: 182 (182-183); 0-12 Months: 363 (363-365)

Supplemental Table 3. Associations of risk factors measured at baseline (2nd trimester of pregnancy) or 9-months postpartum with linear growth at the 6- to 12-month age interval, using a residuals model approach[1]

	Covariates measured in the 2nd trimester of pregnancy			***Covariates measured* at *9* months postpartum**		
	**N**	**Difference in LAZ (95% CI)**	**p**	**N**	**Difference in LAZ (95% CI)**	**p**
Smoking in Household	1070			963		
Never	696	ref	ref	677	ref	ref
Ever	374	-0.02 (-0.09,0.05)	0.529	286	-0.01 (-0.09,0.06)	0.729
Water Treatment	1073			964		
Untreated	585	ref	ref	487	ref	ref
Treated	488	0.04 (-0.03,0.11)	0.266	477	-0.01 (-0.08,0.06)	0.713
Observed Soap	1073			964		
Soap or Detergent	903	ref	ref	858	ref	ref
No Soap	170	0.12 (0.03,0.21)	0.012	106	0.13 (0.02,0.24)	0.021

[1]All models adjusted for the assigned treatment group in the MDIG trial and maternal height, maternal and paternal occupation, maternal and paternal education, and asset index.

Supplemental Table 4. Multivariable-adjusted associations of early postnatal feeding-related factors and household factors with linear growth in five age intervals of infancy in a birth cohort in Dhaka, Bangladesh, based on the ANCOVA modeling approach[1]

	**Age interval in which linear growth outcome was ascertained[2]**														
	0-3 Months			*3-6 Months*			*0-6 Months*			*6-12 Months*			*0-12 Months*		
	**N**	**Difference in LAZ (95% CI)**	**p**	**N**	**Difference in LAZ (95% CI)**	**p**	**N**	**Difference in LAZ (95% CI)**	**p**	**N**	**Difference in LAZ (95% CI)**	**p**	**N**	**Difference in LAZ (95% CI)**	**p**
**Smoking in Household[3]**	1007			1054			1018			1070			1069		
Never	665	ref	ref	687	ref	ref	668	ref	ref	696	ref	ref	697	ref	ref
Ever	342	0.03 (-0.05,0.12)	0.432	367	-0.04 (-0.10,0.03)	0.259	350	-0.03 (-0.13,0.07)	0.547	374	-0.02 (-0.09,0.05)	0.525	372	-0.03 (-0.13,0.08)	0.629
**Water Treatment[3]**	1010			1057			1021			1073			1072		
Untreated	552	ref	ref	576	ref	ref	557	ref	ref	585	ref	ref	580	ref	ref
Treated	458	-0.07 (-0.16,0.01)	0.088	481	0.03 (-0.04,0.09)	0.372	464	-0.04 (-0.14,0.05)	0.360	488	0.04 (-0.03,0.11)	0.243	492	0.03 (-0.08,0.13)	0.616
**Observed Soap[3]**	1010			1057			1021			1073			1072		
Soap or Detergent	848	ref	ref	889	ref	ref	854	ref	ref	903	ref	ref	902	ref	ref
No Soap	162	0.01 (-0.10,0.13)	0.829	168	-0.01 (-0.10,0.08)	0.800	167	-0.02 (-0.15,0.11)	0.781	170	0.12 (0.03,0.21)	0.011	170	0.09 (-0.05,0.23)	0.219
**Breastfeeding Classification 0-3m[4]**	924			922			930			944			977		
EBF	482	ref	ref	474	ref	ref	482	ref	ref	488	ref	ref	510	ref	ref
Predominant	98	0.02 (-0.12,0.16)	0.776	98	-0.01 (-0.12,0.10)	0.842	96	0.0002 (-0.16,0.16)	0.997	98	-0.06 (-0.18,0.05)	0.269	101	-0.06 (-0.24,0.11)	0.486
Partial	315	-0.11 (-0.20,-0.02)	0.019	319	0.06 (-0.02,0.13)	0.138	318	-0.01 (-0.12,0.09)	0.829	322	0.01 (-0.07,0.08)	0.838	330	0.001 (-0.11,0.12)	0.986
None	29	-0.30 (-0.54,-0.07)	0.011	31	-0.03 (-0.21,0.16)	0.771	34	-0.24 (-0.49,0.01)	0.062	36	0.06 (-0.11,0.24)	0.475	36	-0.12 (-0.39,0.16)	0.409
**Breastfeeding Classification 0-6m[4]**				920			936			950			976		
EBF	–	–	–	130	ref	ref	133	ref	ref	135	ref	ref	138	ref	ref
Predominant	–	–	–	72	-0.04 (-0.19,0.10)	0.560	69	-0.06 (-0.27,0.16)	0.608	73	0.05 (-0.10,0.19)	0.548	75	0.01 (-0.23,0.24)	0.960
Partial	–	–	–	643	0.04 (-0.05,0.14)	0.377	654	0.02 (-0.12,0.15)	0.776	659	0.07 (-0.03,0.17)	0.166	680	0.07 (-0.08,0.22)	0.340
None	–	–	–	75	0.03 (-0.12,0.17)	0.734	80	-0.07 (-0.27,0.14)	0.527	83	0.15 (0.004,0.29)	0.044	83	0.07 (-0.16,0.29)	0.563
**EBF Duration (0-3M) (per one month)[4]**	938	0.03 (-0.004,0.07)	0.081	938	-0.01 (-0.04,0.02)	0.359	954	0.002 (-0.04,0.05)	0.917	967	-0.01 (-0.04,0.02)	0.563	1010	-0.01 (-0.06,0.04)	0.707
**EBF Duration (0-6M) (per one month)[4]**	–	–	–	938	-0.01 (-0.03,0.01)	0.254	954	-0.0001 (-0.02,0.02)	0.992	967	-0.01 (-0.02,0.01)	0.486	1010	-0.01 (-0.03,0.02)	0.618
**Animal-source food Exposure (0-3M)[4]**	933			933			944			957			994		
Never	845	Ref	ref	844	ref	ref	854	ref	ref	866	ref	ref	901	ref	ref
Ever	88	-0.14 (-0.27,0.002)	0.054	89	-0.01 (-0.12,0.10)	0.906	90	-0.12 (-0.28,0.04)	0.131	91	0.05 (-0.07,0.16)	0.427	93	-0.07 (-0.25,0.10)	0.416
**Animal-source food Exposure (0-6M)[4]**				929			942			955			983		
Never	–	–	–	637	ref	ref	642	ref	ref	650	ref	ref	669	ref	ref
Ever	–	–	–	292	0.03 (-0.04,0.10)	0.451	300	-0.01 (-0.11,0.09)	0.822	305	0.004 (-0.07,0.08)	0.906	314	-0.02 (-0.13,0.08)	0.661
**Formula Exposure (0-3M)[4]**	934			935			947			960			998		
Never	615	ref	ref	608	ref	ref	620	ref	ref	627	ref	ref	655	ref	ref
Ever	319	-0.13 (-0.22,-0.04)	0.003	327	0.07 (0.01,0.14)	0.034	327	-0.02 (-0.12,0.08)	0.708	333	0.02 (-0.05,0.09)	0.615	343	0.01 (-0.10,0.12)	0.829
**Formula Exposure (0-6M)[4]**				933			947			961			992		
Never	–	–	–	362	ref	ref	364	ref	ref	373	ref	ref	382	ref	ref
Ever	–	–	–	571	0.04 (-0.02,0.11)	0.193	583	0.003 (-0.09,0.10)	0.947	588	0.09 (0.02,0.15)	0.013	610	0.09 (-0.02,0.19)	0.097

[1] All models adjusted for the assigned treatment group in the MDIG trial and maternal height.

[2]Median (IQR) length of time interval in days: 0-3 Months: 90 (90-91), 3-6 Months: 91 (91-91), 0-6 Months: 181 (181-182), 6-12 Months: 182 (182-183), 363 (363-365)

[3]Additionally adjusted for maternal and paternal occupation, maternal and paternal education, and asset index.

[4]Additionally adjusted for neonatal illness, delivery location, delivery mode, maternal and paternal education, asset index, number of children, maternal postnatal BMI, maternal age, infant sex, newborn weight-for-length z-score, and gestational age at birth.

Supplemental Table 5. Multivariable-adjusted associations of early postnatal feeding-related factors and household factors with linear growth in five age intervals of infancy in a birth cohort in Dhaka, Bangladesh, based on the 'change score' modeling approach[1]

	**Age interval in which outcome linear growth was ascertained[2]**														
	0-3 Months			***3-6 Months***			***0-6 Months***			***6-12 Months***			***0-12 Months***		
	**N**	**Difference in LAZ (95% CI)**	**p**	**N**	**Difference in LAZ (95% CI)**	**p**	**N**	**Difference in LAZ (95% CI)**	**p**	**N**	**Difference in LAZ (95% CI)**	**p**	**N**	**Difference in LAZ (95% CI)**	**p**
**Smoking in Household[3]**	1007			1054			1018			1070			1069		
Never	665	ref	ref	687	ref	ref	668	ref	ref	696	ref	ref	697	ref	ref
Ever	342	0.03 (-0.07,0.13)	0.592	367	-0.04 (-0.11,0.02)	0.198	350	-0.03 (-0.14,0.08)	0.603	374	-0.02 (-0.09,0.05)	0.546	372	-0.02 (-0.15,0.10)	0.726
**Water Treatment[3]**	1010			1057			1021			1073			1072		
Untreated	552	ref	ref	576	ref	ref	557	ref	ref	585	ref	ref	580	ref	ref
Treated	458	-0.11 (-0.21,-0.02)	0.022	481	0.03 (-0.04,0.09)	0.420	464	-0.09 (-0.20,0.02)	0.116	488	0.03 (-0.03,0.10)	0.332	492	-0.01 (-0.13,0.12)	0.922
**Observed Soap[3]**	1010			1057			1021			1073			1072		
Soap or Detergent	848	ref	ref	889	ref	ref	854	ref	ref	903	ref	ref	902	ref	ref
No Soap	162	-0.02 (-0.15,0.12)	0.808	168	-0.02 (-0.11,0.07)	0.640	167	-0.05 (-0.20,0.09)	0.474	170	0.11 (0.02,0.21)	0.019	170	0.05 (-0.12,0.22)	0.568
**Breastfeeding Classification 0-3m[4]**	924			922			930			944			977		
EBF	482	ref	ref	474	ref	ref	482	ref	ref	488	ref	ref	510	ref	ref
Predominant	98	0.06 (-0.09,0.22)	0.412	98	-0.001 (-0.11,0.11)	0.987	96	0.05 (-0.13,0.23)	0.570	98	-0.05 (-0.17,0.06)	0.350	101	-0.02 (-0.22,0.18)	0.859
Partial	315	-0.09 (-0.19,0.01)	0.091	319	0.08 (0.004,0.15)	0.040	318	0.004 (-0.12,0.12)	0.950	322	0.01 (-0.06,0.09)	0.701	330	0.03 (-0.10,0.16)	0.645
None	29	-0.26 (-0.53,0.01)	0.055	31	0.02 (-0.17,0.21)	0.863	34	-0.18 (-0.47,0.11)	0.228	36	0.10 (-0.08,0.28)	0.274	36	-0.05 (-0.37,0.27)	0.774
**Breastfeeding Classification 0-6m[4]**				920			936			950			976		
EBF	–	–	–	130	ref	ref	133	ref	ref	135	ref	ref	138	ref	ref
Predominant	–	–	–	72	-0.07 (-0.22,0.08)	0.355	69	-0.13 (-0.37,0.11)	0.302	73	0.03 (-0.12,0.18)	0.704	75	-0.09 (-0.36,0.18)	0.502
Partial	–	–	–	643	0.04 (-0.06,0.14)	0.431	654	0.01 (-0.15,0.16)	0.934	659	0.06 (-0.04,0.16)	0.245	680	0.05 (-0.13,0.22)	0.606
None	–	–	–	75	0.03 (-0.12,0.18)	0.700	80	-0.09 (-0.33,0.14)	0.422	83	0.15 (0.001,0.30)	0.049	83	0.03 (-0.23,0.29)	0.809
**EBF Duration (0-3M) (per one month)[4]**	938	0.03 (-0.02,0.07)	0.238	938	-0.02 (-0.05,0.01)	0.171	954	-0.01 (-0.05,0.04)	0.843	967	-0.01 (-0.04,0.02)	0.457	1010	-0.02 (-0.08,0.03)	0.461
**EBF Duration (0-6M) (per one month)[4]**	–	–	–	938	-0.01 (-0.03,0.004)	0.129	954	-0.003 (-0.03,0.02)	0.808	967	-0.01 (-0.02,0.01)	0.438	1010	-0.01 (-0.04,0.02)	0.468
**Animal-source food Exposure (0-3M)[4]**	933			933			944			957			994		
Never	845	ref	ref	844	ref	ref	854	ref	ref	866	ref	ref	901	ref	ref
Ever	88	-0.05 (-0.21,0.11)	0.513	89	0.04 (-0.08,0.15)	0.537	90	-0.04 (-0.22,0.14)	0.650	91	0.08 (-0.03,0.20)	0.169	93	0.05 (-0.16,0.25)	0.655
**Animal-source food Exposure (0-6M)[4]**				929			942			955			983		
Never	–	–	–	637	ref	ref	642	ref	ref	650	ref	ref	669	ref	ref
Ever	–	–	–	292	0.03 (-0.04,0.10)	0.365	300	-0.01 (-0.13,0.10)	0.803	305	0.005 (-0.07,0.08)	0.896	314	-0.03 (-0.15,0.10)	0.670
**Formula Exposure (0-3M)[4]**	934			935			947			960			998		
Never	615	ref	ref	608	ref	ref	620	ref	ref	627	ref	ref	655	ref	ref
Ever	319	-0.14 (-0.24,-0.04)	0.006	327	0.09 (0.02,0.16)	0.010	327	-0.03 (-0.14,0.09)	0.641	333	0.02 (-0.05,0.09)	0.561	343	0.01 (-0.11,0.14)	0.818
**Formula Exposure (0-6M)[4]**				933			947			961			992		
Never	–	–	–	362	ref	ref	364	ref	ref	373	ref	ref	382	ref	ref
Ever	–	–	–	571	0.06 (-0.01,0.13)	0.093	583	0.02 (-0.08,0.13)	0.660	588	0.09 (0.02,0.16)	0.011	610	0.11 (-0.01,0.23)	0.078

[1]All models adjusted for the assigned treatment group in the MDIG trial and maternal height.
[2]Median (IQR) length of time interval in days: 0-3 Months: 90 (90-91), 3-6 Months: 91 (91-91), 0-6 Months: 181 (181-182), 6-12 Months: 182 (182-183), 363 (363-365)
[3]Additionally adjusted for maternal and paternal occupation, maternal and paternal education, and asset index.
[4]Additionally adjusted for neonatal illness, delivery location, delivery mode, maternal and paternal education, asset index, number of children, maternal postnatal BMI, maternal age, infant sex, newborn weight-for-length z-score, and gestational age at birth.

Supplemental Table 6. Comparison of effect estimates for the associations of feeding-related and household factors with linear growth outcomes in different age intervals of infancy, using five different growth or size modeling approaches, restricted to associations for which there were significant associations using at least one modeling approach.[1]

	**Residuals**	**ANCOVA**	**Change-score**	**Attained size**	**Stunting**
	Difference in LAZ (95% CI)	Difference in LAZ (95% CI)	Difference in LAZ (95% CI)	Difference in LAZ (95% CI)	Relative Risk (95% CI)
**At birth (Size and stunting models)**					
Predominantly breastfed 0-3 months, (compared to exclusive)				-0.09 (-0.29,0.11)	1.66 (1.05,2.62)
No Breastfeeding, 0-3 months (compared to exclusive)	–	–	–	-0.14 (-0.46,0.18)	2.43 (1.20,4.91)
EBF Duration, 0-3 months	–	–	–	0.02 (-0.03,0.08)	0.84 (0.72,0.97)
Animal source foods ever, 0-3 months (compared to never)	–	–	–	-0.23 (-0.43,-0.03)	1.71 (1.11,2.65)
**0 – 3 Months (or at 3 months)**					
Partial Breastfeeding 0-3 months (compared to exclusive)	-0.11 (-0.20,-0.02)	-0.11 (-0.20,-0.02)	-0.09 (-0.19,0.01)	-0.14 (-0.26,-0.02)	2.02 (1.31,3.12)
No Breastfeeding 0-3 months (compared to exclusive)	-0.30 (-0.54,-0.07)	-0.30 (-0.54,-0.07)	-0.26 (-0.53,0.01)	-0.37 (-0.69,-0.05)	2.36 (0.80,6.95)
Animal source foods ever, 0-3 months (compared to never)	-0.13 (-0.27,0.01)	-0.14 (-0.27,0.002)	-0.05 (-0.21,0.11)	-0.28 (-0.46,-0.09)	2.25 (1.38,3.68)
Formula ever, 0-3 months (compared to never)	-0.13 (-0.22,-0.04)	-0.13 (-0.22,-0.04)	-0.14 (-0.24,-0.04)	-0.11 (-0.23,0.01)	1.53 (1.01,2.33)
EBF Duration, 0-3 months	0.03 (-0.005,0.07)	0.03 (-0.004,0.07)	0.03 (-0.02,0.07)	0.05 (-0.005,0.10)	0.74 (0.62,0.89)
EBF Duration, 0-6 months	–	–	–	0.02 (-0.01,0.05)	0.86 (0.77,0.95)
Water Treatment (compared to none)	-0.08 (-0.16,0.01)	-0.07 (-0.16,0.01)	-0.11 (-0.21,-0.02)	-0.01 (-0.12,0.11)	1.17 (0.82,1.68)
**3 – 6 months**					
Partial Breastfeeding 0-3 months (compared to exclusive)	0.06 (-0.01,0.13)	0.06 (-0.02,0.13)	0.08 (0.004,0.15)	–	–
Formula ever, 0-3 months (compared to never)	0.08 (0.01,0.15)	0.07 (0.01,0.14)	0.09 (0.02,0.16)	–	–
**0 – 6 months (or at 6 months)**					
No Breastfeeding 0-3 months (compared to exclusive)	-0.23 (-0.49,0.02)	-0.24 (-0.49,0.01)	-0.18 (-0.47,0.11)	-0.32 (-0.64,-0.01)	2.15 (0.91,5.08)
Animal source foods ever, 0-3 months (compared to never)	-0.11 (-0.27,0.04)	-0.12 (-0.28,0.04)	-0.04 (-0.22,0.14)	-0.23 (-0.42,-0.03)	2.11 (1.28,3.46)
**6 – 12 months**					
No Breastfeeding 0-6 months (compared to exclusive)	0.15 (0.004,0.29)	0.15 (0.004,0.29)	0.15 (0.001,0.30)	–	–
Formula ever, 0-6 months (compared to never)	0.09 (0.02,0.16)	0.09 (0.02,0.15)	0.09 (0.02,0.16)	–	–
No observed soap (compared to observed soap use)	0.12 (0.03,0.21)	0.12 (0.03,0.21)	0.11 (0.02,0.21)	–	–
**0 – 12 months (or at 12 months)**					
Animal source foods ever, 0-3 months (compared to never)	-0.06 (-0.23,0.12)	-0.07 (-0.25,0.10)	0.05 (-0.16,0.25)	-0.19 (-0.39,0.01)	1.62 (1.06,2.47)

[1] Red cells represent statistically significant associations and blue represent non-significant associations whereby $p<0.05$ was considered statistically significant.

Supplemental Table 7. Multivariable-adjusted associations of early postnatal feeding-related factors and household factors with linear growth at four ages of infancy in a birth cohort in Dhaka, Bangladesh, using the attained size modeling approach[1]

	Size at birth			*Attained size at 3 months*			*Attained size at 6 months*			*Attained size at 12 months*		
	**N**	**Difference in LAZ (95% CI)**	**p**	**N**	**Difference in LAZ (95% CI)**	**p**	**N**	**Difference in LAZ (95% CI)**	**p**	**N**	**Difference in LAZ (95% CI)**	**p**
**Smoking in Household[3]**	1093			1007			1018			1069		
Never	717	ref	ref	665	ref	ref	668	ref	ref	697	ref	ref
Ever	376	-0.01 (-0.14,0.11)	0.848	342	0.05 (-0.07,0.17)	0.428	350	-0.03 (-0.15,0.09)	0.627	372	-0.03 (-0.15,0.09)	0.632
**Water Treatment[3]**	1096			1010			1021			1072		
Untreated	592	ref	ref	552	ref	ref	557	ref	ref	580	ref	ref
Treated	504	0.06 (-0.06,0.19)	0.306	458	-0.01 (-0.12,0.11)	0.874	464	0.01 (-0.11,0.13)	0.840	492	0.06 (-0.06,0.18)	0.350
**Observed Soap[3]**	1096			1010			1021			1072		
Soap or Detergent	923	ref	ref	848	ref	ref	854	ref	ref	902	ref	ref
No Soap	173	0.08 (-0.09,0.24)	0.379	162	0.06 (-0.10,0.22)	0.449	167	0.03 (-0.13,0.19)	0.730	170	0.13 (-0.04,0.29)	0.128
**Breastfeeding Classification 0-3m[4]**	982			924			930			977		
EBF	512	ref	ref	482	ref	ref	482	ref	ref	510	ref	ref
Predominant	101	-0.09 (-0.29,0.11)	0.383	98	-0.06 (-0.24,0.13)	0.552	96	-0.07 (-0.26,0.13)	0.494	101	-0.10 (-0.31,0.10)	0.305
Partial	333	-0.06 (-0.19,0.07)	0.368	315	-0.14 (-0.26,-0.02)	0.025	318	-0.03 (-0.16,0.10)	0.632	330	-0.03 (-0.16,0.10)	0.668
None	36	-0.14 (-0.46,0.18)	0.390	29	-0.37 (-0.69,-0.05)	0.023	34	-0.32 (-0.64,-0.01)	0.044	36	-0.19 (-0.51,0.13)	0.255
**Breastfeeding Classification 0-6m[4]**	982			917			936			976		
EBF	138	ref	ref	129	ref	ref	133	ref	ref	138	ref	ref
Predominant	75	0.19 (-0.07,0.46)	0.152	67	0.18 (-0.07,0.43)	0.164	69	0.04 (-0.22,0.31)	0.755	75	0.10 (-0.16,0.37)	0.448
Partial	686	0.06 (-0.12,0.23)	0.517	649	0.02 (-0.14,0.18)	0.804	654	0.04 (-0.13,0.21)	0.664	680	0.10 (-0.07,0.27)	0.255
None	83	0.07 (-0.19,0.33)	0.608	72	-0.05 (-0.30,0.20)	0.686	80	-0.03 (-0.28,0.23)	0.842	83	0.10 (-0.16,0.36)	0.446
**EBF Duration (0-3M) (per one month)[4]**	1016	0.02 (-0.03,0.08)	0.417	938	0.05 (-0.005,0.10)	0.075	954	0.01 (-0.04,0.07)	0.662	1010	0.002 (-0.05,0.06)	0.933
**EBF Duration (0-6M) (per one month)[4]**	1016	0.01 (-0.02,0.04)	0.542	938	0.02 (-0.01,0.05)	0.128	954	0.004 (-0.02,0.03)	0.780	1010	-0.002 (-0.03,0.03)	0.890
**Animal-source food Exposure (0-3M)[4]**	1000			933			944			994		
Never	904	ref	ref	845	ref	ref	854	ref	ref	901	ref	ref
Ever	96	-0.23 (-0.43,-0.03)	0.027	88	-0.28 (-0.46,-0.09)	0.004	90	-0.23 (-0.42,-0.03)	0.023	93	-0.19 (-0.39,0.01)	0.067
**Animal-source food Exposure (0-6M)[4]**	989			928			942			983		
Never	671	ref	ref	637	ref	ref	642	ref	ref	669	ref	ref
Ever	318	0.01 (-0.12,0.14)	0.880	291	-0.06 (-0.18,0.06)	0.304	300	-0.01 (-0.13,0.12)	0.907	314	-0.02 (-0.15,0.10)	0.738
**Formula Exposure (0-3M)[4]**	1004			934			947			998		
Never	658	ref	ref	615	ref	ref	620	ref	ref	655	ref	ref
Ever	346	-0.01 (-0.13,0.12)	0.933	319	-0.11 (-0.23,0.01)	0.062	327	-0.01 (-0.13,0.11)	0.897	343	0.01 (-0.12,0.13)	0.887
**Formula Exposure (0-6M)[4]**	998			930			947			992		
Never	383	ref	ref	355	ref	ref	364	ref	ref	382	ref	ref
Ever	615	-0.04 (-0.16,0.08)	0.499	575	-0.09 (-0.20,0.02)	0.121	583	-0.03 (-0.14,0.09)	0.677	610	0.07 (-0.05,0.19)	0.265

[1]All models adjusted for the assigned treatment group in the MDIG trial and maternal height.
[2]Additionally adjusted for maternal and paternal occupation, maternal and paternal education, and asset index.
[3]Additionally adjusted for neonatal illness, delivery location, delivery mode, maternal and paternal education, asset index, number of children, maternal postnatal BMI, maternal age, infant sex, newborn weight-for-length z-score, and gestational age at birth.

Supplemental Table 8. Multivariable-adjusted associations of early postnatal feeding-related factors and household factors with stunting (length-for-age z-score < -2 SD below the age- and sex-specific median) at four time points[1]

	Stunting at birth			Stunting at 3 months			Stunting at 6 months			Stunting at 12 months		
	N	Relative Risk (95% CI)	p	N	Relative Risk (95% CI)	p	N	Relative Risk (95% CI)	p	N	Relative Risk (95% CI)	p
**Smoking in Household[3]**	1093			1007			1018			1069		
Never	717	ref	ref	665	ref	ref	668	ref	ref	697	ref	ref
Ever	376	1.15 (0.86,1.54)	0.346	342	0.99 (0.70,1.40)	0.943	350	1.16 (0.82,1.63)	0.397	372	0.95 (0.71,1.26)	0.711
**Water Treatment[3]**	1096			1010			1021			1072		
Untreated	592	ref	ref	552	ref	ref	557	ref	ref	580	ref	ref
Treated	504	0.93 (0.70,1.24)	0.616	458	1.17 (0.82,1.68)	0.379	464	1.01 (0.71,1.43)	0.956	492	1.11 (0.84,1.46)	0.478
**Observed Soap[3]**	1096			1010			1021			1072		
Soap or Detergent	923	ref	ref	848	ref	ref	854	ref	ref	902	ref	ref
No Soap	173	1.04 (0.71,1.51)	0.848	162	0.92 (0.58,1.48)	0.737	167	0.91 (0.57,1.45)	0.690	170	0.76 (0.51,1.13)	0.173
**Breastfeeding Classification 0-3m[4]**	982			924			930			977		
EBF	512	ref	ref	482	ref	ref	482	ref	ref	510	ref	ref
Predominant	101	1.66 (1.05,2.62)	0.031	98	1.40 (0.76,2.57)	0.283	96	1.03 (0.56,1.91)	0.927	101	1.05 (0.65,1.70)	0.840
Partial	333	1.41 (0.96,2.06)	0.077	315	2.02 (1.31,3.12)	0.001	318	1.32 (0.86,2.02)	0.210	330	0.98 (0.69,1.37)	0.886
None	36	2.43 (1.20,4.91)	0.013	29	2.36 (0.80,6.95)	0.119	34	2.15 (0.91,5.08)	0.080	36	1.35 (0.61,2.98)	0.457
**Breastfeeding Classification 0-6m[4]**	982			917			936			976		
EBF	138	ref	ref	129	ref	ref	133	ref	ref	138	ref	ref
Predominant	75	0.67 (0.32,1.39)	0.277	67	1.11 (0.44,2.80)	0.826	69	0.82 (0.30,2.19)	0.687	75	0.81 (0.42,1.54)	0.517
Partial	686	0.81 (0.53,1.25)	0.346	649	1.24 (0.64,2.38)	0.527	654	1.21 (0.64,2.26)	0.559	680	0.95 (0.62,1.48)	0.835
None	83	0.93 (0.47,1.84)	0.841	72	1.18 (0.46,3.02)	0.734	80	1.28 (0.54,3.01)	0.578	83	1.09 (0.59,2.03)	0.783
**EBF Duration (0-3M) (per one month)[4]**	1016	0.84 (0.72,0.97)	0.018	938	0.74 (0.62,0.89)	0.001	954	0.89 (0.74,1.07)	0.218	1010	0.96 (0.83,1.11)	0.565
**EBF Duration (0-6M) (per one month)[4]**	1016	0.92 (0.85,1.00)	0.057	938	0.86 (0.77,0.95)	0.004	954	0.95 (0.86,1.04)	0.263	1010	0.98 (0.91,1.06)	0.653
**Animal-source food Exposure (0-3M)[4]**	1000			933			944			994		
Never	904	ref	ref	845	ref	ref	854	ref	ref	901	ref	ref
Ever	96	1.71 (1.11,2.65)	0.016	88	2.25 (1.38,3.68)	0.001	90	2.11 (1.28,3.46)	0.003	93	1.62 (1.06,2.47)	0.027
**Animal-source food Exposure (0-6M)[4]**	989			928			942			983		
Never	671	ref	ref	637	ref	ref	642	ref	ref	669	ref	ref
Ever	318	0.96 (0.68,1.36)	0.825	291	1.22 (0.82,1.82)	0.335	300	1.20 (0.81,1.78)	0.362	314	1.00 (0.73,1.38)	0.982
**Formula Exposure (0-3M)[4]**	1004			934			947			998		
Never	658	ref	ref	615	ref	ref	620	ref	ref	655	ref	ref
Ever	346	1.09 (0.76,1.55)	0.637	319	1.53 (1.01,2.33)	0.047	327	1.13 (0.75,1.71)	0.557	343	0.99 (0.71,1.37)	0.932
**Formula Exposure (0-6M)[4]**	998			930			947			992		
Never	383	ref	ref	355	ref	ref	364	ref	ref	382	ref	ref
Ever	615	1.08 (0.77,1.50)	0.655	575	1.52 (0.98,2.34)	0.059	583	1.41 (0.93,2.13)	0.102	610	1.15 (0.84,1.57)	0.387

[1]All models adjusted for the assigned treatment group in the MDIG trial and maternal height.
[2]Additionally adjusted for maternal and paternal occupation, maternal and paternal education, and asset index.
[3]Additionally adjusted for neonatal illness, delivery location, delivery mode, maternal and paternal education, asset index, number of children, maternal postnatal BMI, maternal age, infant sex, newborn weight-for-length z-score, and gestational age at birth.

Supplemental Table 9. Multivariable association of breastfeeding pattern from 0 to 3 months of age with change in infant length-for-age z-scores in three age intervals and at 12 months of age, using a mixed effects model with linear splines [1,2]

	0-3 Months		***3-6 Months***		***6-12 Months***		***At 12 Months[3]***	
	**Difference in LAZ slope (95% CI)**	**p**	**Difference in LAZ slope (95% CI)**	**p**	**Difference in LAZ slope (95% CI)**	**p**	**Difference in LAZ (95% CI)**	**p**
Breastfeeding Classification 0-3m								
EBF	ref	ref	ref	ref	ref	ref	ref	ref
Predominant	0.03 (-0.13, 0.18)	0.743	-0.03 (-0.16, 0.10)	0.689	-0.07 (-0.20, 0.05)	0.257	-0.16 (-0.40, 0.09)	0.206
Partial	-0.14 (-0.24, -0.04)	0.006	0.13 (0.05, 0.22)	<0.001	0.05 (-0.04, 0.13)	0.281	-0.01 (-0.17, 0.15)	0.883
None	-0.21 (-0.46, 0.05)	0.109	0.06 (-0.15, 0.28)	0.553	0.13 (-0.07, 0.33)	0.188	-0.15 (-0.54, 0.24)	0.444

[1]Mixed effects models were used to model length-for-age z-score as a function of age, with child-specific random intercepts and random slopes. Linear splines were used to capture the age intervals of interest; knots were placed at 91 and 182 days of age (corresponding to 3 and 6 months of age). Interaction terms between breastfeeding pattern from 0-3 months and all spline terms for age were included in the model, and the interaction of breastfeeding pattern with the spline terms are presented in this table. Effect estimates are scaled to reflect the difference in LAZ slope for a 91-day increase in age for the 0-3 month and 3-6 month spline terms and a 182 day increase for the 6-12 month spline term. N= 983 infants contributed at least 1 observation to the model.

[2] Models adjusted for assigned treatment group in the MDIG trial, maternal height, neonatal illness (ever/never), delivery location, delivery type, maternal and paternal education, wealth index, number of children, maternal postnatal BMI, maternal age, infant sex, newborn weight for length z-score, and gestational age at birth.

[3] Marginal effect of breastfeeding pattern from 0-3 months of age on LAZ at 12 months of age was estimated from the mixed effects model with 95% confidence interval

Supplemental Table 10. Sensitivity analyses of associations of early postnatal feeding-related factors with linear growth in four age intervals of infancy using alternative breastfeeding classification schemes, exclusive breastfeeding duration criteria, animal intake criteria and formula intake criteria in the first 6 months of life.[1]

Feeding-related factor (0-6 months)	Age interval in which outcome (linear growth) was ascertained[2]											
	3-6 Months			0-6 Months			6-12 Months			0-12 Months		
	N	Difference in LAZ (95% CI)	p	N	Difference in LAZ (95% CI)	p	N	Difference in LAZ (95% CI)	p	N	Difference in LAZ (95% CI)	p
**Breastfeeding classification alternative A[3]**	920			936			950			976		
EBF	106	ref	ref	110	ref	ref	111	ref	ref	112	ref	ref
Predominant	87	-0.02 (-0.16,0.13)	0.827	83	-0.06 (-0.27,0.15)	0.565	87	0.01 (-0.14,0.15)	0.929	91	-0.02 (-0.24,0.21)	0.890
Partial	652	0.07 (-0.04,0.17)	0.210	663	0.02 (-0.13,0.16)	0.833	669	0.06 (-0.05,0.16)	0.288	690	0.07 (-0.09,0.24)	0.394
None	75	0.05 (-0.10,0.20)	0.542	80	-0.07 (-0.28,0.14)	0.508	83	0.14 (-0.01,0.29)	0.068	83	0.06 (-0.17,0.30)	0.599
**Breastfeeding classification alternative B [4]**	919			935			949			974		
EBF	130	-0.04 (-0.18,0.11)	0.623	133	-0.06 (-0.27,0.15)	0.598	135	0.04 (-0.11,0.19)	0.617	138	-0.003 (-0.23,0.23)	0.977
Predominant	72	0.05 (-0.04,0.14)	0.295	69	0.02 (-0.11,0.16)	0.744	73	0.06 (-0.03,0.16)	0.189	75	0.07 (-0.08,0.22)	0.353
Partial	642	0.03 (-0.11,0.18)	0.674	653	-0.07 (-0.27,0.14)	0.524	658	0.15 (0.005,0.29)	0.043	678	0.06 (-0.16,0.29)	0.591
None	75	ref	ref	80	ref	ref	83	ref	ref	83	ref	ref
**Breastfeeding classification alternative C [5]**	931			947			961			991		
EBF	130	ref	ref	133	ref	ref	135	ref	ref	138	ref	ref
Predominant	73	-0.05 (-0.19,0.10)	0.501	70	-0.07 (-0.28,0.14)	0.528	74	0.03 (-0.12,0.18)	0.697	76	-0.02 (-0.25,0.21)	0.848
Partial	653	0.05 (-0.05,0.14)	0.339	664	0.02 (-0.11,0.16)	0.764	669	0.07 (-0.03,0.17)	0.152	694	0.08 (-0.07,0.23)	0.319
None	75	0.03 (-0.12,0.17)	0.718	80	-0.07 (-0.27,0.14)	0.511	83	0.15 (0.01,0.30)	0.037	83	0.07 (-0.16,0.29)	0.568
**Breastfeeding classification alternative D[6]**	920			936			950			976		
EBF	203	ref	ref	209	ref	ref	214	ref	ref	219	ref	ref
Predominant	72	-0.04 (-0.18,0.09)	0.526	72	-0.18 (-0.38,0.01)	0.062	76	-0.03 (-0.16,0.11)	0.687	77	-0.20 (-0.41,0.01)	0.064
Partial	587	0.03 (-0.05,0.11)	0.396	596	-0.03 (-0.14,0.09)	0.638	598	0.04 (-0.04,0.12)	0.349	618	-0.01 (-0.14,0.11)	0.836
None	58	0.01 (-0.14,0.16)	0.878	59	-0.17 (-0.38,0.04)	0.117	62	0.15 (-0.002,0.30)	0.053	62	-0.04 (-0.27,0.19)	0.742
**Breastfeeding classification alternative E[7]**	920			936			950			976		
EBF	236	ref	ref	240	ref	ref	243	ref	ref	251	ref	ref
Predominant	54	-0.09 (-0.24,0.06)	0.245	54	-0.19 (-0.40,0.03)	0.089	55	-0.04 (-0.19,0.12)	0.637	57	-0.21 (-0.44,0.03)	0.082
Partial	569	0.07 (-0.01,0.14)	0.090	579	0.06 (-0.05,0.17)	0.294	586	0.06 (-0.02,0.14)	0.142	602	0.11 (-0.01,0.23)	0.070
None	61	0.02 (-0.13,0.16)	0.821	63	-0.07 (-0.28,0.13)	0.468	66	0.16 (0.01,0.30)	0.032	66	0.07 (-0.15,0.30)	0.523
**Breastfeeding classification alternative F[8]**	200			197			204			207		
EBF	28	ref	ref	28	ref	ref	28	ref	ref	28	ref	ref
Predominant	18	-0.12 (-0.45,0.20)	0.455	18	-0.26 (-0.67,0.15)	0.215	19	-0.04 (-0.36,0.28)	0.804	19	-0.26 (-0.74,0.22)	0.280
Partial	144	0.03 (-0.18,0.25)	0.770	142	-0.07 (-0.34,0.20)	0.610	146	0.12 (-0.10,0.33)	0.292	149	0.06 (-0.26,0.39)	0.696
None	10	-0.34 (-0.74,0.06)	0.094	9	-0.35 (-0.87,0.18)	0.196	11	0.20 (-0.19,0.58)	0.313	11	-0.23 (-0.80,0.35)	0.440
**Breastfeeding classification alternative G[9]**	891			907			920			941		

Feeding-related factor (0-6 months)	Age interval in which outcome (linear growth) was ascertained[2]											
	3-6 Months			0-6 Months			6-12 Months			0-12 Months		
	N	Difference in LAZ (95% CI)	p	N	Difference in LAZ (95% CI)	p	N	Difference in LAZ (95% CI)	p	N	Difference in LAZ (95% CI)	p
EBF	304	ref	ref	308	ref	ref	311	ref	ref	319	ref	ref
Predominant	47	-0.14 (-0.29,0.02)	0.083	47	-0.25 (-0.48,-0.03)	0.025	48	0.03 (-0.13,0.19)	0.712	49	-0.16 (-0.41,0.08)	0.193
Partial	493	0.06 (-0.01,0.13)	0.110	501	0.03 (-0.07,0.13)	0.581	508	0.03 (-0.04,0.11)	0.392	520	0.08 (-0.03,0.20)	0.167
None	47	-0.01 (-0.17,0.14)	0.889	51	-0.13 (-0.35,0.08)	0.225	53	0.12 (-0.03,0.28)	0.117	53	0.02 (-0.22,0.26)	0.859
**EBF duration alternative A (per one month)[10]**	938	-0.02 (-0.03,-0.001)	0.036	954	0.003 (-0.02,0.02)	0.807	967	-0.01 (-0.02,0.01)	0.379	1010	-0.005 (-0.03,0.02)	0.705
**EBF duration alternative B (per one month)[11]**	938	-0.01 (-0.02,0.01)	0.402	954	0.02 (-0.004,0.04)	0.125	967	-0.004 (-0.02,0.01)	0.595	1010	0.01 (-0.01,0.03)	0.476
**EBF duration alternative C (per one month)[12]**	938	-0.01 (-0.03,0.01)	0.226	954	-0.001 (-0.02,0.02)	0.962	967	-0.01 (-0.02,0.01)	0.468	1010	-0.01 (-0.03,0.02)	0.585
**EBF duration alternative D (per one month)[13]**	938	-0.01 (-0.02,0.01)	0.256	954	-0.0001 (-0.02,0.02)	0.990	967	-0.01 (-0.02,0.01)	0.446	1010	-0.01 (-0.03,0.02)	0.560
**EBF duration alternative E (per one month)[14]**	938	-0.01 (-0.03,0.01)	0.202	954	-0.001 (-0.02,0.02)	0.953	967	-0.01 (-0.02,0.01)	0.491	1010	-0.01 (-0.03,0.02)	0.626
**Animal exposure alternative A[15]**	938			953			967			1009		
Never	646	ref	ref	653	ref	ref	662	ref	ref	695	ref	ref
Ever	292	0.03 (-0.04,0.10)	0.442	300	-0.01 (-0.11,0.09)	0.797	305	0.001 (-0.07,0.07)	0.961	314	-0.03 (-0.14,0.08)	0.577
**Animal exposure alternative B[16]**	929			942			955			983		
Never	619	ref	ref	624	ref	ref	633	ref	ref	652	ref	ref
Ever	310	0.02 (-0.05,0.09)	0.504	318	-0.03 (-0.12,0.07)	0.611	322	0.02 (-0.05,0.09)	0.670	331	-0.03 (-0.13,0.08)	0.647
**Formula exposure alternative[17]**	938			953			967			1009		
Never	367	ref	ref	370	ref	ref	379	ref	ref	399	ref	ref
Ever	571	0.05 (-0.02,0.11)	0.169	583	0.01 (-0.09,0.10)	0.916	588	0.08 (0.02,0.15)	0.014	610	0.09 (-0.02,0.19)	0.105

[1] All models adjusted for the following covariates: assigned treatment group in the MDIG trial, maternal height, neonatal illness (ever/never), delivery location, delivery mode, maternal and paternal education, number of children, maternal postnatal BMI, maternal age, infant sex, newborn weight-for-length z-score, and gestational age at birth.
[2] Median (IQR) duration of interval in days: 0-3 Months: 90 (90-91); 3-6 Months: 91 (91-91); 0-6 Months: 181 (181-182); 6-12 Months: 182 (182-183); 0-12 Months: 363 (363-365)
[3]Breastfeeding classification alternative A is a strict breastfeeding classification, allowing for no exceptions for any exposures other than breastmilk, even if single non-breastmilk exposures occur, at any time
[4]Breastfeeding classification alternative B required that caregivers indicated that the infant was breastfed in both 24-hour recall and 7-day recall questions in order for that week to be classified as exclusively breastfed or predominantly breastfed, and having indicated breastfeeding in at least the 7-day recall for that week to be classified as partially breastfed. This contrasts the primary derivation which allowed caregivers to indicate that the infant was breastfed in the 24-hour recall and/or the 7-day recall in order to be classified as exclusively, predominantly or partially breastfed for that week.
[5]Breastfeeding classification alternative C allowed for missing data in the last 2 weeks before measurement if the infant was already classified as not being exclusively breastfed.
[6] Breastfeeding classification alternative D allows for any 1 week of deviation when classifying feeding pattern
[7] Breastfeeding classification alternative E was based only on last 2 weeks of intake before the end of the 6-month interval
[8] Breastfeeding classification alternative F was based only on infants with no missing breastfeeding data in the first 6 months were included in analyses
[9] Breastfeeding classification alternative G was based on recall of breastfeeding in the 24-hour period preceding the last completed weekly study visit
[10] EBF duration alternative A allowed for up to 1 week of deviation from exclusive breastfeeding to be ignored.
[11] EBF duration alternative B was based on the duration of exclusive breastfeeding following a strict duration classification, allowing for no exceptions for any non-breastmilk exposures, even if single exposures, at any time
[12] EBF duration alternative C required that caregivers indicated that the infant was breastfed in both 24-hour recall and 7-day recall questions in order for that week to count as exclusively breastfed.
[13] EBF duration alternative D is based on the last known week when infants were exclusively breastfed
[14]In EBF duration alternative E, for those infants who were exclusively breastfed until a certain week followed by weeks of missing data, this derivation assumed that EBF continued and the duration of EBF was set to the end of the interval.
[15] In Animal exposure alternative A, less stringent criteria for inclusion in the animal exposure model such that infants who did not have >50% data reported were included in analyses.
[16] In Animal exposure alternative B, infants were classified as having consumed animal products based on mother report during weekly clinical visits and was additionally supplemented with data on animal product consumption collected from mothers retrospectively
[17] In formula exposure alternative, less stringent criteria for inclusion in the formula exposure model such that infants who did not have >50% data reported were included in analyses.

Supplemental Material

Supplemental Table 11. Unadjusted associations of non-modifiable risk factors with linear growth at four ages of infancy in a birth cohort in Dhaka, Bangladesh, using the residuals modeling approach[1]

	**Age interval in which outcome linear growth was ascertained**														
	0-3 Months			***3-6 Months***			***0-6 Months***			***6-12 Months***			***0-12 Months***		
	**N**	**Difference in LAZ (95% CI)**	**p**	**N**	**Difference in LAZ (95% CI)**	**p**	**N**	**Difference in LAZ (95% CI)**	**p**	**N**	**Difference in LAZ (95% CI)**	**p**	**N**	**Difference in LAZ (95% CI)**	**p**
**Infant Sex**	1012			1059			1023			1075			1074		
Boy	513	ref	ref	544	ref	ref	522	ref	ref	547	ref	ref	545	ref	ref
Girl	499	0.17 (0.09,0.25)	0.001	515	0.004 (-0.06,0.07)	0.906	501	0.13 (0.04,0.23)	0.005	528	0.07 (0.002,0.13)	0.044	529	0.17 (0.06,0.27)	0.002
**Birth WAZ**[2]	1010	0.02 (-0.03,0.07)	0.358	1003	0.02 (-0.01,0.06)	0.223	1021	-0.01 (-0.06,0.04)	0.688	1019	-0.01 (-0.04,0.03)	0.785	1072	-0.00 (-0.06,0.06)	0.939
**Birth WFL**[2]	976	0.01 (-0.03,0.05)	0.681	969	-0.02 (-0.06,0.01)	0.147	983	-0.02 (-0.07,0.02)	0.323	983	0.02 (-0.02,0.05)	0.363	1035	0.00 (-0.05,0.06)	0.937
**Location of Delivery**	999			1045			1010			1061			1061		
Hospital/Clinic	867	ref	ref	892	ref	ref	877	ref	ref	906	ref	ref	920	ref	ref
Home	132	-0.08 (-0.20,0.04)	0.190	153	-0.09 (-0.17,0.00)	0.060	133	-0.19 (-0.32,-0.05)	0.008	155	-0.05 (-0.14,0.04)	0.314	141	-0.22 (-0.37,-0.06)	0.006
**Mode of Delivery**	1012			1059			1023			1075			1074		
Vaginal	478	ref	ref	503	ref	ref	484	ref	ref	509	ref	ref	509	ref	ref
Caesarean Section	534	0.04 (-0.04,0.12)	0.366	556	0.03 (-0.03,0.09)	0.373	539	0.04 (-0.05,0.13)	0.394	566	0.01 (-0.05,0.08)	0.694	565	0.06 (-0.05,0.16)	0.284
**Neonatal Hospitalization**[3]	1012			1059			1023			1075			1074		
Never	872	ref	ref	904	ref	ref	873	ref	ref	915	ref	ref	914	ref	ref
Ever	140	0.01 (-0.11,0.13)	0.835	155	0.02 (-0.07,0.10)	0.736	150	0.02 (-0.11,0.15)	0.758	160	0.01 (-0.09,0.10)	0.907	160	0.06 (-0.09,0.20)	0.440
**Maternal Education**	1012			1059			1023			1075			1074		
No Education	46	ref	ref	48	ref	ref	48	ref	ref	46	ref	ref	47	ref	ref
Primary incomplete	207	0.02 (-0.19,0.23)	0.855	224	0.09 (-0.07,0.25)	0.261	212	0.11 (-0.13,0.34)	0.382	227	0.18 (0.01,0.35)	0.038	222	0.29 (0.02,0.57)	0.036
Primary complete	526	0.02 (-0.18,0.22)	0.840	550	0.13 (-0.02,0.28)	0.100	529	0.16 (-0.06,0.39)	0.146	561	0.19 (0.02,0.35)	0.025	560	0.33 (0.07,0.59)	0.012
Secondary complete or higher	233	0.13 (-0.08,0.34)	0.231	237	0.15 (-0.01,0.31)	0.074	234	0.28 (0.05,0.51)	0.019	241	0.30 (0.13,0.47)	0.001	245	0.56 (0.29,0.84)	0.001
**Paternal Education**	941			984			950			997			995		
No education	68	ref	ref	72	ref	ref	70	ref	ref	73	ref	ref	73	ref	ref
Primary incomplete	163	-0.02 (-0.21,0.17)	0.836	171	0.14 (0.001,0.28)	0.049	166	0.17 (-0.04,0.38)	0.104	172	0.10 (-0.05,0.25)	0.191	174	0.27 (0.03,0.50)	0.027
Primary complete	478	0.08 (-0.09,0.24)	0.370	507	0.19 (0.06,0.31)	0.004	484	0.30 (0.11,0.49)	0.002	513	0.18 (0.05,0.32)	0.008	505	0.45 (0.24,0.67)	0.001
Secondary complete or higher	232	0.16 (-0.01,0.34)	0.072	234	0.20 (0.07,0.34)	0.003	230	0.38 (0.18,0.58)	0.001	239	0.29 (0.15,0.44)	0.001	243	0.65 (0.42,0.87)	0.001
**Maternal Occupation**	1012			1059			1023			1075			1074		
Home Maker	944	ref	ref	988	ref	ref	954	ref	ref	1003	ref	ref	1003	ref	ref
Works	68	-0.02 (-0.18,0.15)	0.843	71	-0.09 (-0.21,0.04)	0.159	69	-0.14 (-0.32,0.05)	0.143	72	0.05 (-0.08,0.19)	0.412	71	-0.05 (-0.26,0.16)	0.667
**Paternal Occupation**	1010			1057			1021			1073			1072		
Day laborer/rickshaw drivers/agricultural worker	105	ref	ref	114	ref	ref	109	ref	ref	115	ref	ref	110	ref	ref
Salaried worker	544	0.06 (-0.08,0.20)	0.404	571	0.04 (-0.06,0.15)	0.404	559	0.07 (-0.09,0.22)	0.392	585	0.18 (0.07,0.29)	0.001	590	0.26 (0.08,0.44)	0.004
Private business owner/professional	305	0.06 (-0.08,0.21)	0.389	312	0.05 (-0.06,0.16)	0.373	297	0.13 (-0.04,0.30)	0.122	313	0.19 (0.08,0.31)	0.001	315	0.32 (0.13,0.51)	0.001
Jobless	19	0.02 (-0.30,0.35)	0.884	19	0.03 (-0.22,0.28)	0.826	18	0.01 (-0.37,0.39)	0.960	19	0.31 (0.04,0.57)	0.023	18	0.30 (-0.13,0.74)	0.171
Other	37	0.01 (-0.24,0.25)	0.963	41	0.11 (-0.07,0.30)	0.238	38	0.13 (-0.15,0.40)	0.376	41	0.24 (0.04,0.43)	0.017	39	0.35 (0.03,0.67)	0.031
**Number of Siblings**	1012			1059			1023			1075			1074		
No siblings	467	ref	ref	487	ref	ref	476	ref	ref	493	ref	ref	499	ref	ref
1 sibling	398	0.01 (-0.08,0.10)	0.777	420	0.01 (-0.06,0.07)	0.847	400	-0.01 (-0.11,0.09)	0.781	429	-0.06 (-0.13,0.01)	0.093	419	-0.08 (-0.20,0.03)	0.151
2 or more siblings	147	-0.16 (-0.28,-0.03)	0.012	152	0.01 (-0.08,0.11)	0.792	147	-0.18 (-0.32,-0.04)	0.013	153	-0.09 (-0.19,0.01)	0.075	156	-0.25 (-0.41,-0.10)	0.002
**Wealth Index**	1012	0.02 (-0.005,0.04)	0.115	1059	0.01 (-0.01,0.03)	0.276	1023	0.03 (0.004,0.06)	0.025	1075	0.06 (0.04,0.08)	0.001	1074	0.09 (0.06,0.12)	0.001
**Maternal Age**	1012	-0.01 (-0.02,-0.004)	0.006	1059	0.01 (0.003,0.02)	0.008	1023	-0.005 (-0.02,0.01)	0.412	1075	0.004 (-0.003,0.01)	0.262	1074	0.0003 (-0.01,0.01)	0.954
**Maternal BMI**[2,4]	971	-0.01 (-0.02,-0.003)	0.010	1021	0.01 (-0.001,0.01)	0.105	990	-0.01 (-0.02,0.003)	0.140	1056	0.01 (0.004,0.02)	0.004	1046	0.00 (-0.01,0.02)	0.512
**Maternal Height**	1012	0.02 (0.01,0.02)	0.001	1059	0.01 (0.01,0.02)	0.001	1023	0.03 (0.02,0.04)	0.001	1075	0.01 (0.01,0.02)	0.001	1074	0.04 (0.03,0.05)	0.001

[1] Non-modifiable factors refer to those that are not modifiable or not immediately modifiable in the postnatal period
[2] Weight-for-age Z-scores, WAZ; Weight-for-length, WFL; Body mass index, BMI
[3] Hospitalization within the first 28 days of life
[4] Measured at 12-months postpartum, as a proxy for preconception BMI

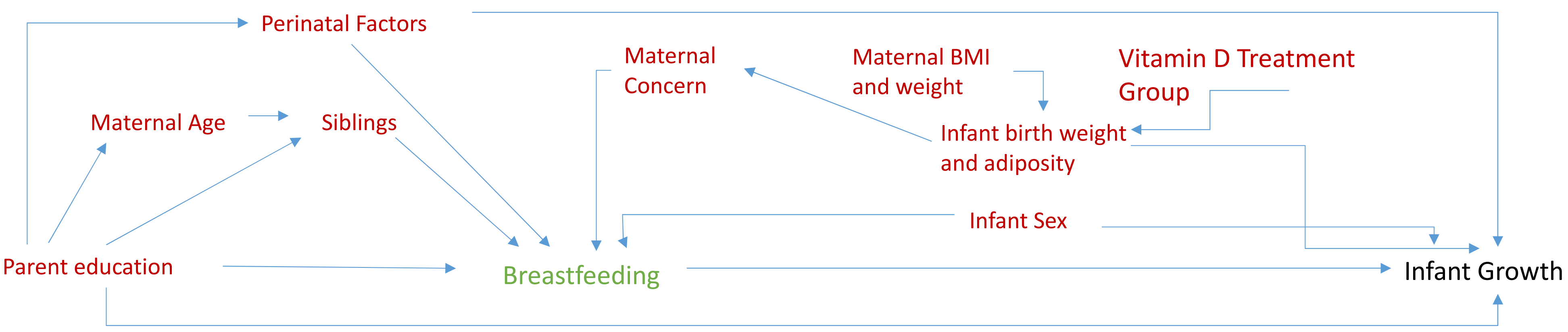

Supplemental Figure 1: Conceptual model depicting breastfeeding as exposure of interest related to infant growth. Factors in red are hypothesized confounders.

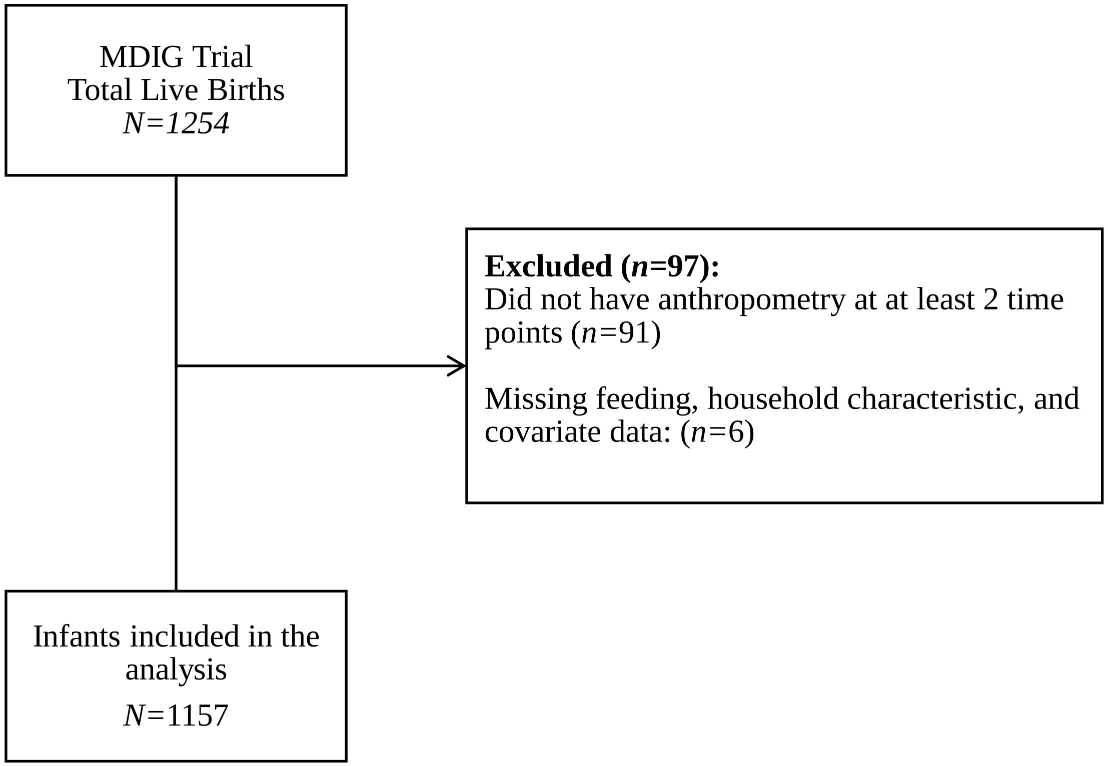

Supplemental Figure 2: Participant eligibility flow diagram.

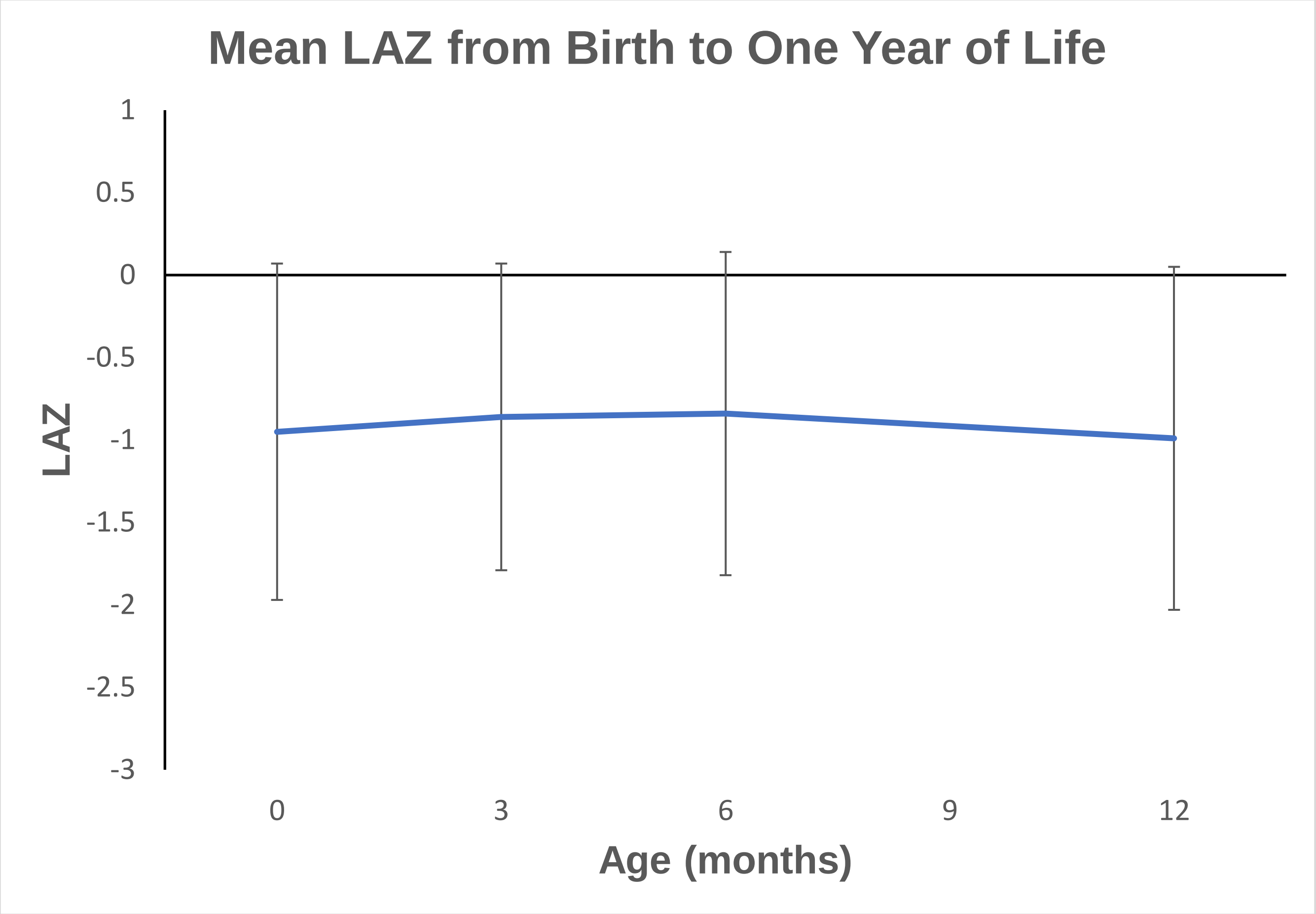

Supplemental Figure 3: Average length-for-age z-score trajectory from birth to twelve months of life. Vertical lines represent one standard deviation above or below the mean LAZ (blue line) at each time point: birth: -0.95 (1.02), 3 months: -0.87 (0.93), 6 months: -0.84 (0.98), 12 months: -1.00 (1.04). The median age in days (25th percentile-75th percentile) at each time point was 1 day (1-3) at birth, 91 days (91-92) at 3 months, 182 days (182-183) at 6 months, and 365 days (365-367) at 12 months.

Supplemental References

1. World Health Organization. Indicators for assessing infant and young child feeding practices: Part 1 Definitions. Conclusions of a consensus meeting held 6–8 November 2007 in Washington D.C., USA. Geneva: World Health Organization; 2008.
